# A Comprehensive Review of Non-Covalent Radiofluorination Approaches Using Aluminum [^18^F]fluoride: Will [^18^F]AlF Replace ^68^Ga for Metal Chelate Labeling?

**DOI:** 10.3390/molecules24162866

**Published:** 2019-08-07

**Authors:** Cyril Fersing, Ahlem Bouhlel, Christophe Cantelli, Philippe Garrigue, Vincent Lisowski, Benjamin Guillet

**Affiliations:** 1 Institut de Recherche en Cancérologie de Montpellier (IRCM), University of Montpellier, INSERM U1194, Montpellier Cancer Institute (ICM), 34298 Montpellier, France; 2 Nuclear Medicine Department, Montpellier Cancer Institute (ICM), University of Montpellier, 208 Avenue des Apothicaires, 34298 Montpellier CEDEX 5, France; 3 CERIMED, Aix-Marseille University, 13005 Marseille, France; 4 Centre de recherche en CardioVasculaire et Nutrition (C2VN), Aix-Marseille University, INSERM 1263, INRA 1260, 13385 Marseille, France; 5 Institut des Biomolécules Max Mousseron, UMR 5247, CNRS, Université de Montpellier, ENSCM, UFR des Sciences Pharmaceutiques et Biologiques, 34093 Montpellier CEDEX, France; 6 Department of Nuclear Medicine, Aix-Marseille University, Assistance Publique-Hôpitaux de Marseille (AP-HM), 13385 Marseille, France

**Keywords:** radiofluorination, aluminum fluoride, coordination chemistry, bifunctional chelating agent, NOTA, PET, molecular imaging

## Abstract

Due to its ideal physical properties, fluorine-18 turns out to be a key radionuclide for positron emission tomography (PET) imaging, for both preclinical and clinical applications. However, usual biomolecules radiofluorination procedures require the formation of covalent bonds with fluorinated prosthetic groups. This drawback makes radiofluorination impractical for routine radiolabeling, gallium-68 appearing to be much more convenient for the labeling of chelator-bearing PET probes. In response to this limitation, a recent expansion of the ^18^F chemical toolbox gave aluminum [^18^F]fluoride chemistry a real prominence since the late 2000s. This approach is based on the formation of an [^18^F][AlF]^2+^ cation, complexed with a 9-membered cyclic chelator such as NOTA, NODA or their analogs. Allowing a one-step radiofluorination in an aqueous medium, this technique combines fluorine-18 and non-covalent radiolabeling with the advantage of being very easy to implement. Since its first reports, [^18^F]AlF radiolabeling approach has been applied to a wide variety of potential PET imaging vectors, whether of peptidic, proteic, or small molecule structure. Most of these [^18^F]AlF-labeled tracers showed promising preclinical results and have reached the clinical evaluation stage for some of them. The aim of this report is to provide a comprehensive overview of [^18^F]AlF labeling applications through a description of the various [^18^F]AlF-labeled conjugates, from their radiosynthesis to their evaluation as PET imaging agents.

## 1. Introduction

### 1.1. ^68^Ga in PET Imaging and Radiolabeling: Pros and Cons

**Figure 1:**
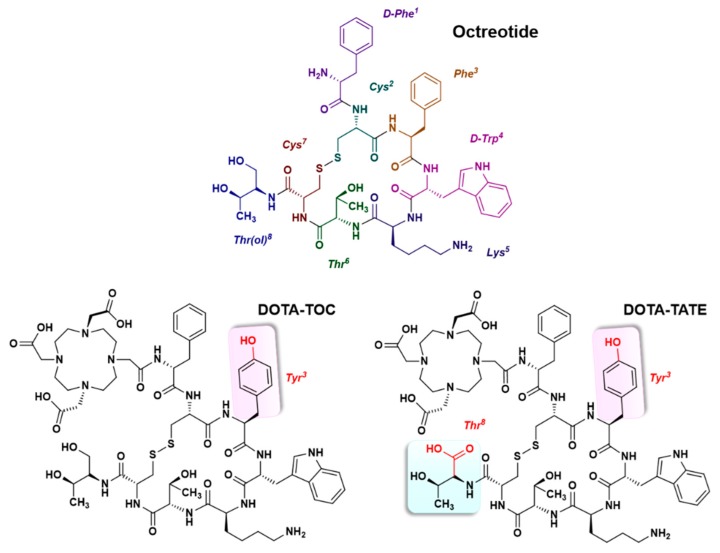
Chemical structures of somatostatin analogs octreotide, DOTA-TOC, and DOTA-TATE.

Positron emission tomography (PET) is currently an essential medical imaging technique in the diagnosis of cancer diseases, therapeutic efficacy monitoring, preclinical research and general molecular imaging [1]. The most widely used PET imaging radiopharmaceutical in current clinical practice is 2-deoxy-2-[^18^F]fluoro-d-glucose or [^18^F]fluorodeoxyglucose ([^18^F]FDG), a glucose analog whose hydroxyl group at position 2 is replaced by a [^18^F]fluorine atom. In recent years, the chemistry of another β+ emitting radioelement, gallium-68, has also acquired a renewed interest by its application in PET imaging [2,3]. ^68^Ga can be obtained from a long life ^68^Ge/^68^Ga generator (^68^Ge half-life = 271 days) which makes it readily available [4,5], relatively inexpensive and independent of a cyclotron or a reactor production. Thus, like ^99m^Tc in single-photon emission computed tomography (SPECT), the use of ^68^Ga in PET imaging is made possible in every hospital [6]. ^68^Ga has a rather short half-life (68 min), consistent with the relatively fast mechanism of distribution, metabolism and elimination from the blood compartment of small bioconjugated peptides, such as somatostatin analogs (Figure 1). Thereby, compared to ^111^In, ^68^Ga allows the realization of imaging examinations in 1–3 h and reduces irradiation of patients by a factor of 2 to 3 [7]. Significant progress in ^68^Ga coordination chemistry has been made in recent years, motivated by the success and promising nature of the first radiopharmaceuticals using this radiometal, in particular [^68^Ga]Ga-DOTA-TOC, first ^68^Ga-labeled somatostatin analog [8,9], ^68^Ga radiolabeling of DOTA-TATE quickly followed [10].

^68^Ga can form stable complexes with many chelators, especially polyaminocarboxylates such as DOTA, NOTA, and their derivatives [11]. NOTA effectively complexes ^68^Ga under relatively mild labeling conditions, while forming a [^68^Ga]Ga-DOTA complex requires a ~100 °C heating step that may be incompatible with some vector molecule.

However, despite the advantages of ^68^Ga like its obtaining from a generator or its metallic character that allows labeling of very diverse molecules by coordination chemistry [4,12], fluorine-18 still occupies a privileged place among the radionuclides used in PET imaging [13]. This is particularly related to its better dosimetry profile and almost ideal physical properties for this type of application (Table 1) [14]. Although the most used ^18^F-labeled radiopharmaceutical in Nuclear Medicine is [^18^F]FDG, a growing number of new imaging agents containing this radioelement have recently been licensed or are currently in clinical trials [15]. Whereas ^18^F is not the most suitable radioisotope for imaging all types of cancer or molecular targets [16,17], several peptide receptors such as integrins, somatostatin, or bombesin would be ideal targets for a fluorinated radiotracer [18]. Compared to ^18^F, the proportion of positrons emitted by ^68^Ga is lower (around 88% decay for ^68^Ga versus 97% for ^18^F), in favor of more electronic capture transformation. Furthermore, the mean free path of gallium-68 positrons in tissues is relatively significant because of their high maximum energy. These factors inherent to the radionuclide, associated with different physical properties related to PET technology [19], cause so-called partial volume effects [20,21], designating effects of blur and spurious signals appearing on PET images and related to a lack of spatial resolution.

These disadvantages are particularly encountered in small animal imaging by ^68^Ga-labeled tracers [22,23]. Indeed, because of the high maximum energy of gallium-68 positrons, the spatial location of the annihilation point is generally quite distant from the emission point. Before annihilation, positron travels a random path, deviated from its initial trajectory by inelastic diffusions. As a result, ^68^Ga PET imaging has a lower sensitivity and a lower spatial resolution than ^18^F PET, with suboptimal quantification properties [24]. Nevertheless, these drawbacks can somehow be compensated by the high contrast achieved with several ^68^Ga-radiotracers that display a significant target specificity compared to [^18^F]FDG, in the absence of resulting background noise.

### 1.2. The Place of ^18^F in Routine Radiolabeling

In contrast, fluorine-18 PET imaging provides very good spatial resolution and sensitivity, partially offsetting the loss of contrast due to non-specific tissue uptake.

**Figure 2:**
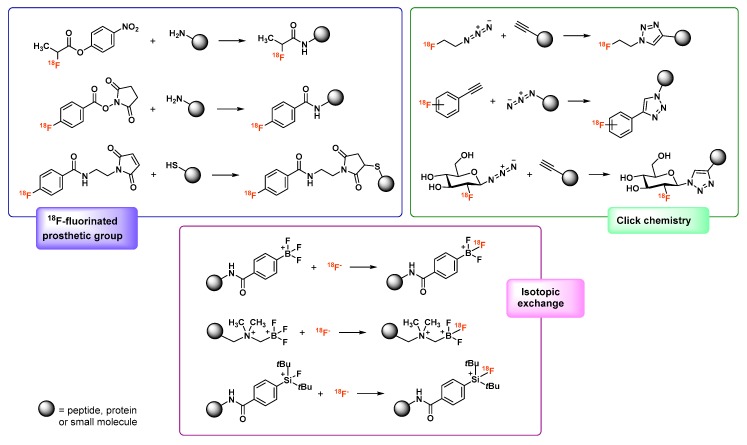
Examples of radiofluorination techniques using substitution or addition reactions, click chemistry or isotopic exchange.

Regarding the radiolabeling step, the non-metallic nature of fluorine makes it incompatible with coordination chemistry approaches used with gallium and requires covalent radiolabeling [25]. In small molecules, ^18^F is generally bonded to a carbon atom of the original structure. To radiolabel peptides or proteins, ^18^F is brought within a prosthetic group, which is then coupled to the vector molecule. Isotope exchange methods using silyl [26,27], phosphorous or boron-containing derivatives [28,29,30] also represent attractive alternatives [31,32,33]. Covalent ^18^F-radiolabeling of a molecule is a multi-step process [34] that may involve quite drastic reaction conditions (use of anhydrous organic solvents, heating at high temperatures). This type of protocol usually starts with trapping ^18^F on an anion-exchange cartridge, then elution of pure concentrated [^18^F]fluoride. It is then dried by heating under inert atmosphere and solubilized in the reaction solvent [35]. [^18^F]fluoride species is finally used to substitute a leaving group present on the precursor via nucleophilic substitution. The radiolabeled intermediate is then purified, most often by high-performance liquid chromatography (HPLC) and in the case of a prosthetic group, it can finally be bonded to the vector of interest though a reaction depending on its structure [25]: acylation, alkylation, nucleophilic addition or click chemistry, for example (Figure 2).

After this covalent radiolabeling step, the fluorinated molecule has also to undergo a purification step. The entire process usually lasts 1–3 h, making it too long, restrictive, and not reliable enough for use in everyday practice. Therefore, the development of a simple and rapid radiolabeling method for ^18^F-fluorination of imaging vectors would be of great interest in the development of new radiopharmaceutical candidates. Non-covalent radiofluorination by complexation of aluminum [^18^F]fluoride (Al[^18^F]F), deriving from isotopic exchange techniques, seems to meet this need [36].

### 1.3. Non-Covalent Radiofluorination Using Aluminum [^18^F]fluoride

In view of the constraints related to covalent radiofluorination methods [37,38], a new ^18^F-labeling strategy for molecules conjugated to a bifunctional chelating agent has recently been described. Its principle is based on the strength of the bond between fluoride anion ([^18^F]F^−^) and aluminum cation (Al^3+^). The resulting salt tends to form thermodynamically stable and kinetically inert metal chelates with polyaminocarboxylate ligands.

Within the periodic table of elements, aluminum belongs to group 13 (atoms with a ns^2^ np^1^ valence electron shell that can easily lose three electrons to form trivalent cations). It is the third chemical element and the most abundant metal in Earth’s crust (about 9%) [39]. As a hard metal ion [40], the most stable oxidation state of aluminum, Al^3+^, strongly interacts with hard bases [41] and, thus, easily complexes with O^2−^ and F^−^ to form very stable structures like alumina (Al_2_O_3_) or cryolite (Na_3_AlF_6_). Indeed, its small effective ionic radius (~50 pm) makes the aluminum ion highly electropositive and not easily polarizable. In aqueous solution, Al^3+^ ions also tend to form mono- then polyhydroxylated species which precipitate at pH 5–9. In a very basic solution, Al(OH)_3_ precipitates resolubilize to form aluminates (like AlO_2_^−^, for example).

The binding of aluminum to its ligands is partially covalent and essentially involves ionic or even electrostatic interactions. The most stable aluminum complexes are obtained with multidentate ligands with negatively charged oxygen donor sites (alkoxides or carboxylates, for example). The affinity of these ligands increases linearly with the basicity of their donor sites [42,43], supporting the essentially ionic character of their interaction with Al^3+^.

Aluminum forms more stable complexes with fluorine than with other halogens, with aluminum-fluorine bond strength of approximately 670 kJ/mol [44,45] (compared to the C-F bond of fluoromethane, having a force of 485 kJ/mol [46]). Among all the metallic species, Al^3+^ is only surpassed by the scandium cation Sc^3+^, which forms with fluorides even stronger bonds [47]. Al-F bond is, moreover, very stable in vivo, making small amounts of aluminum fluoride complex compatible with biological systems [48,49]. With a maximum coordination number of 6, the Al^3+^ ion can be complexed by a suitable chelator and form, in the presence of fluoride ions, a ternary complex [fluorine- aluminum-chelator]. It preferentially adopts an octahedral geometry if the valency of the ligand allows it.

**Figure 3:**
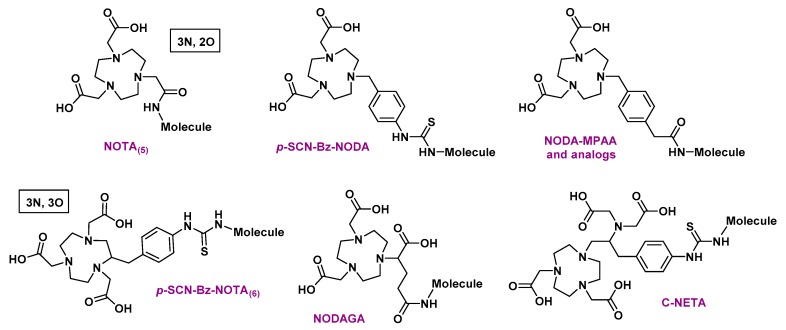
Examples of pentadentate or hexadentate bifunctionnal chelators used to radiolabel molecules with [^18^F]AlF.

One of the most essential points in the formation of complexes with aluminum fluoride is the choice of a suitable chelator, with the ability to remain stable for several hours in biological media. Since aluminum preferentially forms octahedral complexes, a pentadentate ligand would be needed, leaving a free binding site for the fluoride ion. Nevertheless, beyond these considerations, the first chelation studies of [^18^F]AlF began with the use of DTPA [50], a heptadentate linear chelator known to form stable complexes with other group 13 atoms such as indium [51]. In these experiments, pH appeared as a critical parameter for the formation of aluminum monofluoride (AlF)^2+^. If the pH is too basic, metal tends to bond with a hydroxyl anion instead of fluoride and precipitates [43]. If the pH is too acidic, the favored fluorinated species would be hydrogen fluoride (HF) [52]. Therefore, a pH close to 4 would favor the formation of aluminum monofluoride while also being adapted to the complexation of this salt with a polyaminocarboxylate chelating agent [53]. A ternary complex of DTPA-conjugated peptide, [^18^F]F^-^ and Al^3+^ could have thus been obtained in more than 90% yield, by mixing these three species in a heated pH 4 buffer solution. This complex has, however, not shown sufficient stability in water and serum [50]. NOTA, known to form stable complexes with Al^3+^ was then tried: [^18^F]AlF-labeled NOTA-peptide isolated yields were quite low (5–20% yield), but the complex was stable in serum during 4 h at 37 °C. Attempts to form [^18^F]AlF complexes with larger macrocyclic chelators like DOTA have also been undertaken but final coupling yields were rather disappointing [54]. It has finally been demonstrated that a better labeling efficiency was obtained, for example, with NODA-MPAA, Bz-NODA, or pentavalent NOTA analogs (written NOTA_(5)_). This is correlated to the « 3N, 2O » configuration adopted by the donor atoms of the ligand. It facilitates the formation of stable octahedral aluminum complexes while a unique metallic coordination site is kept free for fluoride fixation [55]. To the contrary, the « 3N, 3O » configuration of hexavalent NOTA derivatives (written NOTA_(6)_) creates a favorable environment for the formation of stable chelates with aluminum alone. Indeed, the presence of a third carboxylate group in NOTA_(6)_ interferes with the coordination of fluoride with aluminum [56]. A stable binding of aluminum fluoride to a chelator is therefore greatly influenced by the structure of the latter. To date, most [^18^F]AlF -labeled molecules contain a triazacyclononane-derived chelator (Figure 3).

The purpose of this review is to provide a comprehensive overview of the use of the [^18^F]AlF methodology to radiolabel different type of compounds such as peptides, proteins or small molecules. The radiolabeling, purification and analytical conditions will be discussed, as well as the preclinical study of the radiofluorinated conjugates. Lastly, the [^18^F]AlF-labeled molecules already evaluated in humans will be reported.

## 2. Overview of [^18^F]AlF-Labeled Molecules Conjugated to Chelating Agents

**Figure 4:**
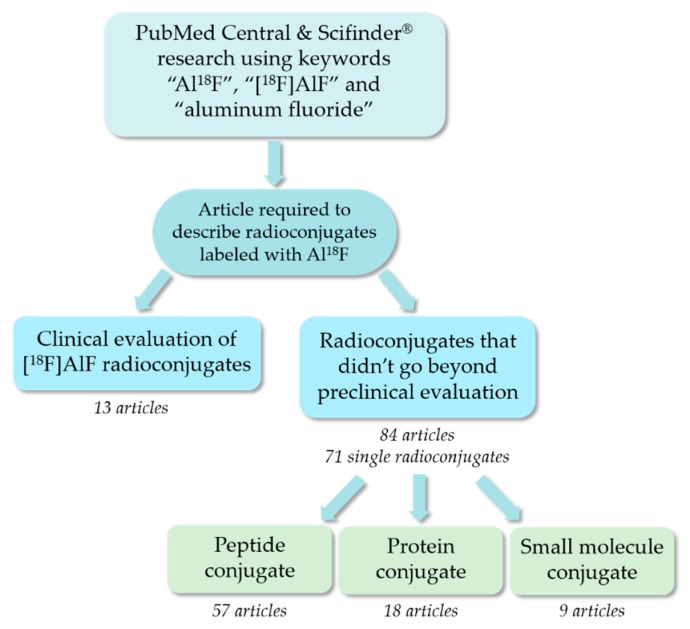
Process of article selection for this review.

Non-covalent radiofluorination using [^18^F]AlF, published for the first time by W. J. McBride in 2009 [50], has taken a substantial and rapid extent since the bases of this radiochemistry concept were presented, leading today to first clinical PET imaging applications. Several types of vectors have been used as substrates of this radiolabeling technique: peptides, proteins such as antibodies and related, or even small molecules. For this review, articles were searched using the databases PubMed Central and Scifinder^®^ with the latest search date of February 2019. The keywords “Al^18^F”, “[^18^F]AlF” and “aluminum fluoride” were entered in the search engines. The titles of the articles were reviewed to determine if the work described radioconjugates labeled with [^18^F]AlF. Based on this, more than a hundred articles were chosen, then articles displaying exclusively clinical evaluations were discarded at first. Eighty-four articles about [^18^F]AlF-radioconjugates that did not go beyond preclinical evaluation were retained and read in depth, corresponding to a total of 71 single radioconjugates. These 84 articles were classified depending on the nature of the conjugate (peptide, protein or small molecule, see Figure 4). An electronic standardized data collection table has then been used to gather (when informed) several data concerning each article: nature of the radiolabeled compound and chelator, molecular target of the radioconjugate, animal model used, radiolabeling, purification and analysis conditions, preparation time, radiolabeling yield, molar activity obtained, and in vitro stability of the final compound (see Supplementary material).

### 2.1. [^18^F]AlF-Labeled Peptide Conjugates

#### 2.1.1. Development of the [^18^F]AlF-Labeling Methodology

**Figure 5:**
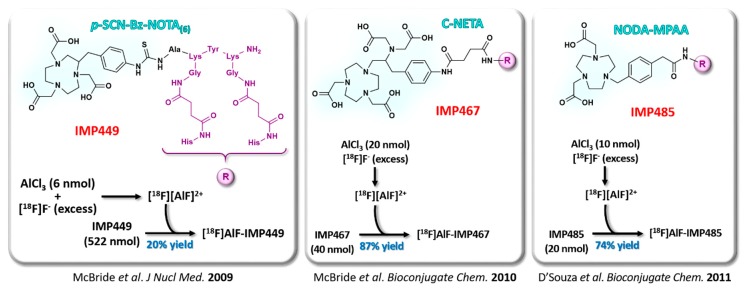
Structure and simplified radiofluorination protocols of the first [^18^F]AlF-labeled peptides.

As previously stated, peptide bioconjugates are particularly suitable for [^18^F]AlF radiolabeling, the rather rapid in vivo pharmacokinetics of these compounds being compatible with fluorine-18 half-life. The first peptide studied in this context is a hapten, IMP449, used in pretargeting approaches of carcinoembryonic antigen (CEA) with a bispecific antibody [50]. This peptide was initially conjugated to NOTA_(6)_, its DTPA-conjugated analogs (IMP272 and IMP375) showing insufficient stability both in water and in human serum. Radiolabeling attempt involved a large excess of a peptide (522 nmol IMP449 for 6 nmol AlCl_3_, thus 87 equiv.). To demonstrate the versatility of this new radiolabeling approach, NOTA_(6)_ has first been replaced by C-NETA (IMP467) with promising results [55]. This experiment also highlighted the interest of a moderated peptide excess (only 2 equiv.) for labeling efficiency. Good labeling yields, reaching 74%, have also been achieved using a NODA-MPAA-conjugate (IMP485) (Figure 5), a pentavalent chelator better suited to [^18^F]AlF [57]. Subsequently, the same research team verified the possibility of transposing this method to other vectors by successfully applying it to a NOTA_(5)_-octreotide derivative [58]. The biodistribution study of [^18^F]AlF-labeled NOTA_(5)_-octreotide showed an identical profile to that of its ^68^Ga-labeled analog, confirming that this fluorination protocol did not affect the in vivo biological properties of the conjugate. Optimization of NOTA_(5)_-octreotide radiofluorination with [^18^F]AlF was the first work to show the favorable influence of the ionic strength on the labeling efficiency through the addition of a hydrophilic organic co-solvent (like acetonitrile or ethanol) to the reaction buffer [59]. Peptide concentration also appeared as an essential parameter for a high yielded labeling, the best results obtained with approximately 200 µM of NOTA_(5)_-octreotide in a ~300 µL total reaction volume.

With the identification of NODA and NOTA_(5)_ analogs as preferential chelators for an easy and high-yielded radiolabeling, efforts were then concentrated on the development of a labeling protocol applicable to a NOTA_(5)_-peptide lyophilisate formulated in a kit [60]. In the example of IMP485, the formulation contained peptide (20 nmol, nearly 2 equiv.), AlCl_3_ (12 nmol, 1 equiv.), ascorbic acid and potassium hydrogen phthalate as buffers and α,α-trehalose dehydrate as a filling agent. [^18^F]F^-^ was added in a mixture of NaCl 0.9% and ethanol (1:1; 400 µL total volume). After heating at 110 °C for 15 min, the radioconjugate was purified on hydrophilic-lipophilic balance (HLB) or alumina cartridge. The [^18^F]F^−^ quality seemed to significantly influence the labeling effectiveness: indeed, fluorine-18 solution directly recovered from the cyclotron outlet contains different contaminants (essentially metallic traces [61]) and therefore needs to be purified before being used to form [^18^F]AlF. The use of radiopharmaceutical grade sodium [^18^F]fluoride ([^18^F]NaF), indicated for bone imaging, can also be an alternative, although expensive. Contrary to the [^18^F]fluoride directly isolated from the cyclotron, this product already passed through a cation exchange separation step and an anion exchange cartridge purification.

These preliminary studies, having shown the good biodistribution profile of [^18^F]AlF-IMP467 [57] and the in vivo stability of [^18^F]AlF-IMP485 [60], confirmed both the feasibility of biomolecules radiolabeling by [^18^F]AlF, the potential of these compounds as specific imaging agents, and the possibility of their formulation as a kit in the perspective of a clinical use. The interest of fluorine-18 over gallium-68 was also quickly supported by a study comparing preclinical imaging properties of [^18^F]AlF-IMP449 and [^68^Ga]Ga-IMP228 (its DOTA-conjugated analog). Pretargeted immunoPET images showed the same intensity in the tumor for the two radiotracers, but the resolution of ^18^F images was better than the ^68^Ga images [62].

Many other target-specific biomolecules have subsequently been radiolabeled with [^18^F]AlF and studied at a preclinical stage. The most frequently performed tests include the evaluation of in vitro target binding, plasma stability, in vivo biodistribution, and PET imaging in murine xenograft models. Global data from these preclinical studies suggest that most of these radiolabeled biomolecules remain intact during their uptake, distribution, and elimination. 

#### 2.1.2. [^18^F]AlF-Labeled Peptides for Integrins Receptors Imaging

The α_V_β_3_ integrin receptor plays a key role in the regulation of angiogenesis, an essential function in the growth and metastasis of many types of cancer [63,64]. To target integrin α_V_β_3_ receptor and image tumor angiogenesis, several probes based on the arginine-glycine-aspartic acid (RGD) amino acid motif were developed, some having been evaluated through clinical trials [65].

Dimeric cyclic RGD peptides have been particularly studied as potential PET imaging agents. Due to the laborious radiofluorination procedures used to label these vectors, the [^18^F]AlF-fluorination strategy appeared as a very appealing way to simplify this critical step. Liu et al. first described in 2011 the synthesis of an [^18^F]AlF-NOTA_(5)_-RGD_2_ radioconjugate that showed a good in vitro serum and in vivo stability [66]. Compared to [^18^F]F-FP-RGD_2_, its analog labeled with a ^18^F-fluorinated prosthetic group (four-steps, two-pot radiosynthesis, 2 h reaction time), [^18^F]AlF-NOTA_(5)_-RGD_2_ production was more efficient (one step radiosynthesis, 40 min total time) for similar biodistribution and imaging properties. 

**Figure 6:**
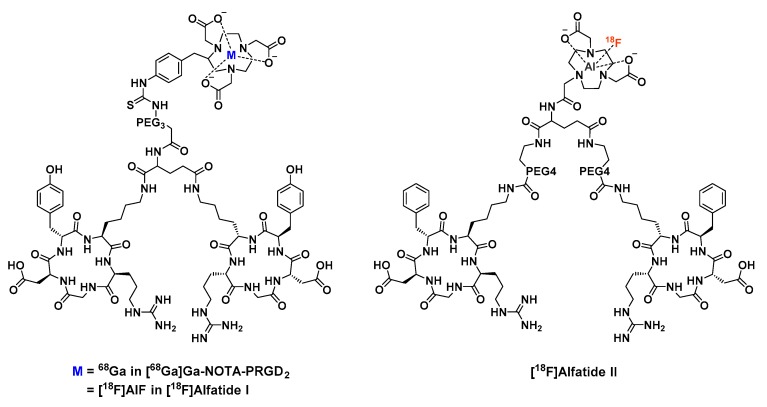
Chemical structures of three dimeric arginine-glycine-aspartic acid (RGD) peptides.

The addition of a PEG_3_ chain between *p*-SCN-Bn-NOTA_(6)_ and the glutamic acid motif that links the two RGD monomers led to NOTA-PRGD_2_, also called alfatide I when radiolabeled with [^18^F]AlF (Figure 6). PET imaging properties of this compound were compared to those of its ^68^Ga-labelled analog in U87MG-xenografted mice, only showing a slightly higher tumor uptake for alfatide I [67]. In the same publication, Lang et al. interestingly specify that whether [^18^F]F^−^ was purified and concentrated on an exchange ions column or used directly from the cyclotron target, the final labeling yields were not affected, contrary to the statement of McBride [57].

On the same U87MG human glioblastoma tumor model, Guo et al. quantitatively compared the pharmacokinetic parameters of alfatide I, its covalently-radiofluorinated analog [^18^F]FPPRGD_2_ and [^68^Ga]Ga-NOTA-PRGD_2_ [68]. No significant difference was found among those three RGD peptide radiotracers, though alfatide I showed a slightly higher binding potential and specific distribution volume in the tumor.

Other applications for [^18^F]AlF-NOTA_(6)_-PRGD_2_ were investigated with convincing results, like the longitudinal visualization of ischemia/reperfusion-induced myocardial angiogenesis in rats [69], or the tumor angiogenesis detection associated with the early therapeutic efficacy monitoring of antiangiogenic therapy in a human nasopharyngeal carcinoma xenograft model [70].

The type of chelator used for RDG dimers functionalization has also been modulated: Dijkgraff et al. synthesized a NODAGA-RGD_2_ derivative that could be labeled with [^18^F]AlF, ^68^Ga or ^111^In [71]. Due to its « 3N, 3O » configuration can saturate aluminum coordination sphere, NODAGA is not the most suitable chelator for [^18^F]AlF, resulting in only modest labeling efficacy (20% yield). However, the hexadentate geometry of NODAGA fits very well with gallium and indium (82% and 91% labeling yield respectively). The use of multiple isotopes allows images comparison from SPECT and PET, biodistribution study of these three radioconjugates showing comparable results for each.

Lang et al. explored the stability of the glutamic acid link between the two RGD monomers [72] and stated that the existence of a free α-amine is at risk of increasing the dimer hydrolysis. Formation of a thiourea linkage with this α-amine is also an instability factor (particularly in acidic conditions and at high temperature), justifying the role of the PEG motif in alfatide I. However, thiourea still showed sensitivity to radiolysis, especially in the absence of ethanol. 

Imaging properties of alfatide I were also studied on epidermoid lung carcinoma [73], alveolar adenocarcinoma and prostate cancer animal models [74], concomitantly with its most recent evaluations in human (see Section 3).

Influence of the PEG chain on the labeling process and pharmacokinetics of RGD2 probes was studied as well and was found to have no significant impact on the labeling yield [75]. In the three compounds evaluated by Guo et al., NOTA_(5)_ was linked to the dimer by a carboxamide bond (to avoid oxidation of thiourea motif) and tyrosine of the cyclic RGD part was replaced by a phenylalanine that could not be oxidized when heating (Figure 6). [^18^F]AlF-labeled NOTA_(5)_-E(PEG_4_-cRDGfK)_2_ (later called alfatide II) showed the highest in vitro receptor binding affinity, along with the lower liver uptake and higher tumor accumulation. The two PEG_4_ linkers between each RGD and the central glutamic acid may provide a proper distance between the two cyclic motifs that could enable their simultaneous integrin binding.

After its identification as a promising alternative for PET imaging of α_V_β_3_ integrin receptor expression, alfatide II was compared to [^18^F]FDG for parametric monitoring of tumor therapy response to doxorubicin and abraxane through a dual tracer imaging approach [76]. With a more significant variation of the alfatide II binding potential values than that of [^18^F]FDG, either doxorubicin or abraxane treatments seemed to imply more changes in tumor angiogenesis than metabolism. Besides, quantitative kinetic parameters calculated from dynamic data of the dual-tracer single-scan imaging were more sensitive than static imaging.

Alfatide II was also evaluated on muscular inflammation [77], epidermoid lung carcinoma [78] and glioblastoma mouse models [79], in parallel with first administrations in man.

In addition to dimeric cyclic RGD peptides, a simple monomeric NODA-SCN-RGD conjugate was synthesized by Shetty et al. [80] and labeled with [^18^F]AlF. Although not directly compared in this study, biodistribution profile and PET imaging results for this monomer were very similar to those of ^18^F-labeled RGD dimers [66,67]. It’s worth mentioning that the automated radiolabeling procedure of a similar NOTA_(5)_-RGD molecule has been described on two different platforms (GE TRACERlab FX_FN_ and Trasis AllInOne) and is suitable for accessing radioconjugates with high radiochemical purity [81].

**Figure 7:**
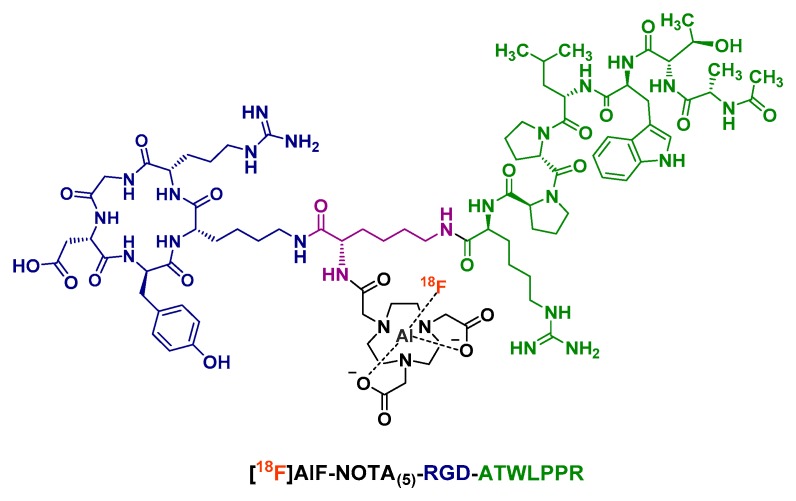
Chemical structure of [^18^F]AlF-NOTA_(5)_-RGD-ATWLPPR heterodimeric radioconjugate.

In addition to integrin α_V_β_3_, neuropilin-1 (NRP-1) is a functional regulator that interacts with the vascular endothelial growth factor (VEGF) receptor and is particularly expressed in tumor angiogenic vessels [82]. The ATWLPPR heptapeptide showed high binding specificity to NRP-1 but a relatively low tumor accumulation [83,84]. Hence, Wu et al. designed an [^18^F]AlF-labeled NOTA_(5)_-RGD-ATWLPPR heterodimeric radioconjugate that was studied in vivo on U87MG tumor-bearing mice [85]. Due to the shortness of the lysine linker between the two peptidic motifs (Figure 7), this heterodimer did not display enhanced binding affinity, only a better tumor targeting efficacy. Tumor uptake and tumor-to-organ ratios of [^18^F]AlF-RGD-ATWLPPR were higher than those of its monomeric [^18^F]AlF-labeled analogs. Interestingly, blocking studies showed that the RGD motif of the heterodimer could still bind to its target when NRP-1 was blocked and likewise for the ATWLPPR part after α_V_β_3_ was blocked. However, PET imaging of [^18^F]AlF-NOTA_(5)_-RGD-ATWLPPR highlighted a bone uptake, suggesting a lack of in vivo metabolic stability for this tracer.

Different integrins than α_V_β_3_ can be targeted by specific peptide probes for cancer-related imaging. It’s the case of integrin α_V_β_6_, for which Hausner et al. have synthesized [^18^F]AlF-NOTA_(5)_-PEG_28_-A20FMDV2 as an ^18^F-fluorinated radiotracer [86]. Despite its rather long linear amino acid sequence (20 residues) and its PEG_28_ chain, the radiotracer did not show sensitivity to the relatively harsh reaction conditions (100 °C, 15 min). Interestingly, the organic co-solvent used for the radiolabeling step is dimethylsulfoxide (DMSO) and not ethanol or acetonitrile as in most of the other protocols. Compared to its covalently radiofluorinated analog [87], [^18^F]AlF-NOTA_(5)_-PEG_28_-A20FMDV2 showed an increased renal uptake and retention, probably due to its functionalization with NOTA_(5)_ [88]. Thus, the pharmacokinetic profile of this radiotracer needs to be improved and emphasized the potential influence of a polyanionic metal chelator on physicochemical properties of a bioconjugate.

#### 2.1.3. [^18^F]AlF-Labeled Peptides for PSMA Imaging

Prostate-specific membrane antigen (PSMA) is a transmembrane glycoprotein with a glutamate carboxypeptidase activity and is significantly overexpressed in nearly all prostate cancers [89]. This malignancy being the most common worldwide in men [90], efficient radiotracers for accurate staging in primary prostate cancer and localization of early recurrences are needed. PSMA radioconjugates are currently intensively investigated in the clinical field, both as diagnostic and therapeutic agents [91]. For PET imaging, several radiofluorinated PSMA ligands obtained via [^18^F]AlF chelation were described, with a particular interest in the nature of the chelating part.

**Figure 8:**
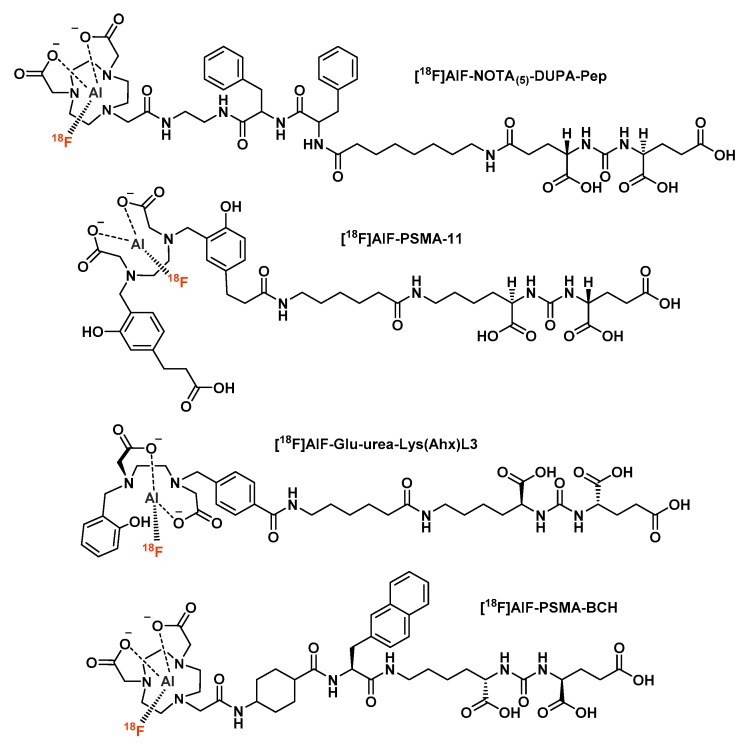
Chemical structures of several [^18^F]AlF-labeled PSMA ligands.

Malik et al. were the first to synthesize a glutamate-urea conjugate bearing a NOTA_(5)_ ring to be labeled with [^18^F]AlF [92]. This PSMA radioligand, called [^18^F]AlF-NOTA_(5)_-DUPA-Pep (Figure 8), was obtained in good radiochemical yields (83 ± 1.1%). In the same way as most of the previously commented protocols, these radiochemical yields (RCY) were reached when the molar ratio of Al^3+^ to peptide was 0.15. However, neither in vitro stability data nor in vivo studies were available for this compound.

Due to its high stability constant for the Ga^3+^ cation [93], acyclic chelator *N*′*N*-bis(2-hydroxybenzyl)ethylendiamin-*N*,*N*′-diacetic acid (HBED) was used in replacement of nine-membered cyclic chelators to complex [^18^F]AlF. Malik et al. described the radiofluorination of a PSMA-HBED conjugate, later called PSMA-11 (Figure 8) [94]. Labeling conditions involved 26 nmol of peptide and 30 nmol of AlCl_3_. Interestingly, the authors highlighted the significant decomposition of the radiocomplex at high temperatures and the very good radiochemical yields that could be achieved by lowering the reaction heating (79% RCY within 1 min at 30 °C), thanks to HBED kinetics properties. Moreover, [^18^F]AlF-PSMA-HBED displayed excellent serum stability for 4 h and a promising binding coefficient to its target (K_D_ = 10.3 nM versus 12.58 nM for [^68^Ga]Ga-PSMA-HBED).

Afterward, a first preclinical evaluation of [^18^F]AlF-PSMA-11 has been proposed [95]. In addition to a fast-renal clearance associated with high kidney and bladder uptake, biodistribution study of [^18^F]AlF-PSMA-11 by microPET imaging of non-grafted C57BL/6 mice showed a slight bone uptake, probably related to a limited in vivo defluorination. Dosimetric calculations extrapolated absorbed doses for [^18^F]AlF-PSMA-11 in human from mouse PET biodistribution data and pointed out kidneys as the dose-limiting organ, the most conservative calculations predicting the maximum administered human activity limit to be 564 MBq. These results remain encouraging for the feasibility of [^18^F]AlF-PSMA-11 clinical translation.

In this perspective, improved radiofluorination methods of [^18^F]AlF-PSMA-11 were described, applicable for clinical routine use in compliance with GMP conditions. Al-Momani et al. validated a manual preparation process of [^18^F]AlF-PSMA-11 under classical reaction conditions (radiolabeling in sodium acetate buffer 0.5 M pH 4.2 containing [^18^F]F^-^ with 50% *v/v* ethanol, 22 nmol PSMA-11 and 30 nmol AlCl_3_, heated at 50 °C for 15 min), ended by a purification on HLB cartridge and a filtration through a 0.22 µm sterile filter. The final [^18^F]AlF-PSMA-11 solution in 1% ethanol/saline remained of high radiochemical purity (RCP) after 4 h (>98%). The pH of this solution was 6.5 and its sterility was confirmed, along with the absence of bacterial endotoxins (< 5 EU/mL), making this [^18^F]AlF-PSMA-11 solution injectable according to the European Pharmacopoeia criteria. This radiotracer was also compared in vitro with four of its ^68^Ga-labeled analogs on LNCaP and 22RV1 cell lines: [^18^F]AlF-PSMA-11 displayed higher cell uptake than three of its analogs and showed no degradation in human serum.

Automated radiosynthesis methods for [^18^F]AlF-labeling of PSMA-11 are another important argument in favor of the clinical translation of this tracer. Kersemans et al. proposed the optimization of a SynthraFCHOL module to obtain [^18^F]AlF-PSMA-11 within 35 min in relatively modest yields (21 ± 3%) but with a high molar activity (120 ± 28 GBq/µmol, superior to that of [^68^Ga]Ga-PSMA-11 [96]) and quality controls in accordance with the European Pharmacopeia guidelines, allowing large scale applications of this tracer, e.g., clinical trials [97]. The authors emphasized the importance of the sequence in which the reagents are added, with the need of [^18^F]F^−^ incubation with Al^3+^ prior to addition of PSMA-11 to maintain reproducible and sufficient yields. They also point out the fluoride purification step by quaternary methylamine (QMA) cartridge and the use of metallic sharps in the automated system as factors limiting higher labeling yields.

Giglio et al. optimized the synthesis of [^18^F]AlF-PSMA-11 on a Tracerlab FXFN^®^ (GE) platform and obtained USP-grade radiotracer in about 18% yield, with a molar activity up to 544 GBq/µmol [98]. The optimized labeling conditions involved only 60 µg peptide, compared to 200 µg used in Kersemans protocol [97]. The final product was stable in its formulation vial for 4 h (radiochemical purity >90%) whereas its stability in human plasma was maintained only during 1 h, forcing a short PET images acquisition time after IV administration.

Eventually, Cleeren et al. reported the design of an original aliphatic chelator called L3, that displayed high complexation yields with [^18^F]AlF (up to 95%) at moderate temperature (40 °C heating, versus 100–120 °C for NOTA and NODA bioconjugates) and demonstrated high in vivo stability with no significant bone uptake [99]. As a proof of concept, L3 was conjugated with the Glu-Urea-Lys pattern then radiolabeled with [^18^F]AlF (Figure 8). Like the radiolabeled chelator alone, [^18^F]AlF-Glu-urea-Lys-(Ahx)L3 was highly stable in vivo in healthy mice, despite a fast clearance from plasma. The main interest of the L3 complexing agent is its chelation with [^18^F]AlF at ambient temperature and thus its compatibility with heat-sensitive biomolecules; this will be discussed in detail in the [^18^F]AlF-labeled protein conjugates section.

A comparative study between two PSMA-11 derivatives has recently been conducted by Lütje et al. [100]. In contrast to [^68^Ga]Ga-PSMA-11, [^18^F]AlF-PSMA-11 appeared to be unstable in water (64.5% radiochemical purity immediately after purification and 52.7% at 2 h post-purification) but remained relatively stable in 25 mM NH_4_OAc pH 6.9 (94.7% radiochemical purity at 2 h post-purification). In vivo, a significant signal after injection of [^18^F]AlF-PSMA-11 in LS174T-PSMA-xenografted mice was observed in the bones, which is in line with the in vitro stability results. Although only minor interferences originate from this bone accumulation on microPET/CT imaging, this localization could hamper the visualization of small metastatic lesions in patients. Besides, biodistribution also demonstrated fast renal uptake of both compounds. However, [^18^F]AlF-PSMA-11 uptake was significantly lower compared to that of [^68^Ga]Ga-PSMA-11 (43.5% ID/g and 105.8% ID/g at 2 h p.i., respectively). Both on visual non-quantitative analysis and quantitative analysis of PET/CT images acquired at 1 h p.i., clearest visualization of PSMA-expressing tumors was obtained with [^18^F]AlF-PSMA-11.

Recently, Liu et al. reported a novel [^18^F]AlF-labeled PSMA ligand, [^18^F]AlF-PSMA-BCH (Figure 8), evaluated through a preclinical study and a pilot clinical study on 11 newly diagnosed prostate cancer patients [101]. [^18^F]AlF-PSMA-BCH was synthesized in modest RCY (32.2 ± 4.5%), with moderate molar activity (13.2 to 18.9 GBq/µmol, that could probably be increased by automated synthesis) but with a high radiochemical purity (>99%). The radioconjugate is highly hydrophilic (logP = −2.76 ± 0.01), PSMA-specific and stable both in vitro and in vivo. With a usual accumulation profile in LNCaP tumor bearing mice, [^18^F]AlF-PSMA-BCH did not cause any signs of radiotoxicity in animals. In patients, this radiotracer showed a good tolerance profile, associated with an intense accumulation in the kidney as well as submandibular, parotid and lacrimal glands. Considering organ radiation dosimetry, kidneys were the most critical organs (0.135 ± 0.003 mGy/MBq), followed by salivary glands, spleen and liver. Each of the 11 patients had at least one observable tumor lesion, for a total of 37 tumor lesions observed. As all lesions visualized at 1 h p.i. were also visible at 2 h p.i. with a SUVmax and contrast higher on the late images of most patients, imaging at a later timepoint may be a better option. This pilot study of [^18^F]AlF-PSMA-BCH demonstrated its good imaging properties for prostate cancer and allows to consider this radiotracer for further extended clinical studies.

#### 2.1.4. [^18^F]AlF-Labeled Peptides for Gastrin-Releasing Peptide Receptor Imaging

In a similar way to PSMA, gastrin-releasing peptide receptor (GRPR) is also overexpressed in prostatic tumor cells while only low levels of receptors are found in normal prostate tissue [102,103]. Due to the poor in vivo stability of the 27-mer mammalian gastrin-releasing peptide (GRP), known to be a GRPR-binding ligand, its stable 14-amino acid amphibian peptide analog bombesin (BBN) has generated many efforts in the development of original bioconjugates for GRPR targeting [104,105]. Currently, several BBN-like peptides have been bioconjugated and labeled with photon-emitting radionuclides (for SPECT or PET imaging) or particle-emitting radioisotopes in a targeted radionuclide therapy perspective.

**Figure 9:**
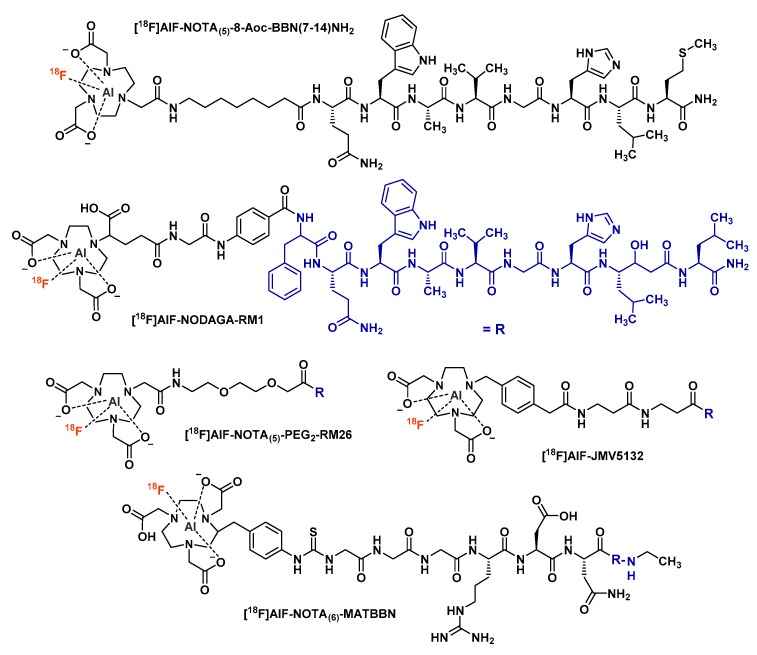
Chemical structures of several [^18^F]AlF-labeled bombesin (BBN) analogs.

The first attempt to radiolabel a BBN analog with [^18^F]AlF was reported by Dijkgraaf et al. in collaboration with the team of W. J. McBride [106]. In this study, [^18^F]AlF and ^68^Ga radiolabeling of NOTA_(5)_-8-Aoc-BBN(7-14)NH_2_ (8-Aoc = 8-aminooctanoic acid) (Figure 9) are compared, along with the in vitro and in vivo properties of the two radiopeptidoconjugates. The ^18^F-labeled peptide showed a slightly higher lipophilicity than its ^68^Ga-labeled analog (logP = −1.47 and −1.98, respectively), the two radiotracers being rapidly cleared from the blood (< 0.07%ID/g at 1 h post-injection in PC-3-xenografted mice). [^18^F]AlF-NOTA_(5)_-8-Aoc-BBN(7-14)NH_2_ displayed a moderately higher uptake in PC-3 tumors than its ^68^Ga-labeled equivalent (2.15 and 1.24%ID/g at 1 h p.i., respectively), the same profile also being more pronounced for pancreas uptake (27.09 and 5.93%ID/g, respectively). Overall, the biodistribution of these two radiopeptides was not statistically different, indicating that the labeling technique did not affect the in vivo fate of the BBN derivative. This work therefore presented [^18^F]AlF-labeled BBN analogs as suitable tracers for in vivo GRPR imaging.

With the same perspective, Liu et al. compared two [^18^F]AlF-labeled BBN-related conjugates, NODAGA-RM1 (Figure 9) and NODAGA-AMBA, to their ^64^Cu-labeled analogs [107]. Although the ^64^Cu-labeled radioconjugates could be synthesized in high yields (>85%), the « 3N, 3O » configuration of NODAGA is not optimal for [^18^F]AlF chelation. The fluorinated NODAGA-RM1 and NODAGA-AMBA derivatives were therefore obtained in quite poor yields (5.6% and 4.9%, respectively). However, a purification step by semi-preparative HPLC allowed the final radiopeptides to reach high radiochemical purity (>95%) and sufficient molar activities (>1.85 GBq/µmol). In further studies, [^18^F]AlF- and [^64^Cu]Cu-NODAGA-RM1 tended to be more stable in mouse serum (>90% after 1 h), with a more favorable in vivo retention in PC-3 tumors and imaging quality. They were also considered better than [^18^F]AlF-NOTA_(5)_-8-Aoc-BBN(7-14)NH_2_.

Due to the favorable properties this vector displayed for GRPR expression imaging [108], Varasteh et al. studied the [^18^F]AlF-labeling of NOTA_(5)_-PEG_2_-RM26 (Figure 9), an antagonist analog of bombesin containing a diethylene glycol (PEG_2_) spacer [109]. Obtained in good labeling yields (60–65%) and with high molar activity (55 GBq/µmol), this radioconjugate remained stable both in vitro and in vivo. In an in vitro cellular uptake assay, [^18^F]AlF-NOTA_(5)_-PEG_2_-RM26 incubated with PC-3 cells showed a slow internalization with only 14% of the cell-associated radioactivity after 4 h of incubation corresponding to the internalized activity. This could be explained by the antagonistic function of RM26. However, GRPR antagonists tend to be considered superior to agonists for tumor imaging, with favorable in vivo characteristics [110]. The NOTA_(5)_-PEG_2_-RM26 vector demonstrated a specific uptake in PSMA-positive organs (e.g., pancreas, stomach and small intestine), more pronounced for the [^18^F]AlF-labeled molecule than its ^68^Ga-labeled analog. Unfortunately, no comparison was made with non-PEGylated derivatives. These global findings suggest that [^18^F]AlF-NOTA_(5)_-PEG_2_-RM26 could be a suitable alternative to its ^68^Ga-labeled analog for clinical application.

An alternative linker modulation is proposed by Pan et al. through the study of [^18^F]AlF-NOTA_(6)_-MATBBN (Figure 9), a BBN antagonist analog with a hydrophilic peptidic linker (GGGRDN) [111]. Despite the non-optimal valency of the chelator (« 3N, 3O » configuration) for aluminum fluoride, radiolabeling of this conjugate was completed within only 30 min, with good yields (62.5 ± 2.1%). LogP value for this [^18^F]AlF-labeled BBN analog was −2.40 ± 0.07, slightly lower than that of [^18^F]AlF-NOTA_(5)_-8-Aoc-BBN(7-14)NH_2_ (−1.47 ± 0.05), probably because of the peptide spacer. Hence, modified in vivo pharmacokinetics could be attributed to this parameter. For example, in vivo PET quantification experiments revealed a good tumor uptake at 1 h p.i. for [^18^F]AlF-NOTA_(6)_-MATBBN, higher than that of other [^18^F]AlF-labeled BBN analogs [106]. This vector also showed significant in vivo superiority over [^18^F]FDG in terms of specific tumor uptake in PC-3 tumor-bearing mice and tumor-to-blood or tumor-to-muscles uptake ratios. Moreover, the PC-3 tumor uptake of [^18^F]AlF-NOTA_(6)_-MATBBN was significantly higher than that of its covalently fluorinated analog [^18^F]FP-MATBBN, positioning the [^18^F]AlF-labeled derivative as a compatible PET tracer candidate for clinical evaluations in prostate cancer cells PET imaging.

In a comparative approach, Chatalic et al. opposed [^18^F]AlF-labeled NODA conjugate GRPR antagonist JMV5132 (Figure 9) to both its ^68^Ga-labeled NODA and DOTA-conjugated analogs [112]. [^18^F]AlF-JMV5132 could be obtained in very good yield (88%) in only 20 min, with a quite good molar activity (40 ± 4 GBq/µmol) and without the need for purification by solid phase extraction. Considering [^18^F]AlF- and [^68^Ga]Ga-JMV5132, these two radiopeptidoconjugates displayed comparable logP values (−1.56 and −1.40, respectively), in vivo biodistribution and pharmacokinetic characteristics and PC-3 tumor uptake values (4.96 and 4.73%ID/g at 1 h p.i., respectively). PET images obtained with [^18^F]AlF-JMV5132 showed higher spatial resolution than those obtained with the ^68^Ga-labeled tracers, most likely because of the longer positron range of gallium-68 compared to fluorine-18. Overall, with a favorable tumor and organs uptake profile, [^18^F]AlF-JMV5132 showed improved imaging properties, compared with the previously reported [^18^F]AlF-NOTA_(5)_-8-Aoc-BBN(7-14)NH_2_ GRPR agonist [106].

Carlucci et al. studied the [^18^F]AlF-labeling along with usual in vitro and in vivo properties of two lanthionine-stabilized BBN analogs [113]. These peptidoconjugates contain thioether cross-linked amino acids that confer resistance to peptidases through the high stability of this bond [114,115]. Bearing a NODAGA-like chelator, the two conjugates considered here were radiolabeled within 1 h in modest to good yields (around 60%), using a large amount of acetonitrile in the reaction medium (about 80% of the final reaction volume). [^18^F]AlF-labeled 4,7-lanthionine-BBN and its 2,6-derivative are both highly hydrophilic (logD = −2.14 and −2.34, respectively) and displayed an improved affinity for their molecular target compared to the same non-conjugated peptides. Similar findings were also observed by Varasteh et al. with the NOTA_(5)_-PEG_2_-RM26 ligand [108]. In vitro, stability of both tracers is quite good in NaCl 0.9% (>90% after 4 h) but considerably lower in human plasma (>75% after 4 h). In vivo, the average fraction of intact tracer in the tumor area was around 88% at 1 h p.i., suggesting a positive influence of the lanthionine stabilization on the biological half-life of these tracers.

#### 2.1.5. [^18^F]AlF-Labeled Peptides for Other Molecular Targets Imaging in Oncology

Many pathologically upregulated physiological processes can differentiate a tumor from normal tissue, thus providing a wide range of prospective molecular targets for imaging probes. Since they mostly display proper in vivo kinetics, metabolic stability, and especially a good tolerance towards bulky modifications, peptides offer a maximum degree of freedom and flexibility. They can easily be conjugated to a chelating agent (with or without a spacer) and radiolabeled without losing their targeting properties. Consequently, and because of the convenience offered by [^18^F]AlF-labeling protocols, several other peptidic tracers were labeled with this method and reported as potential radiotracers for cancer PET imaging.

**Figure 10:**
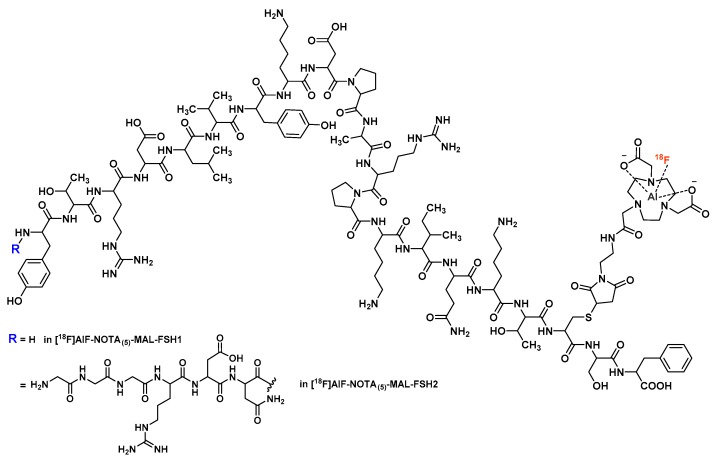
Chemical structures of [^18^F]AlF-NOTA_(5)_-MAL-FSH1 and [^18^F]AlF-NOTA_(5)_-MAL-FSH2.

Targeting follicle-stimulating hormone receptor (FSHr)-overexpressing tumors [116,117], Xu et al. proposed an [^18^F]AlF-labeled NOTA_(5)_-maleimide conjugate of FSH1 [118], a linear 21-mer peptide (Figure 10) [119]. Obtained within 30 min in moderate RCY (48.6 ± 2.1%) but with a sufficient molar activity (>30 GBq/µmol), [^18^F]AlF-NOTA_(5)_-MAL-FSH1 displayed a favorable in vitro retention in PC-3 cells, along with a persistent in vivo tumor accumulation in PC-3-xenografted mice. However, suboptimal pharmacokinetics (especially high abdominal uptake) could hamper its potential clinical applications.

To overcome this drawback, the same research team introduced a hydrophilic linker (GGGRDN) to the FSH1 peptide, now called FSH2 (Figure 10) [120]. Indeed, this peptide sequence has been confirmed to efficiently improve the in vivo imaging properties of other radiopeptidoconjugates [111,121]. [^18^F]AlF-NOTA_(5)_-MAL-FSH2 were obtained in similar labeling yields than its FSH1 analog (41.46 ± 10.36% versus 48.6 ± 2.1%, respectively) and displayed a better anti-FSHr IC_50_ value (103 ± 1.12 nM versus 252 ± 1.12 nM, respectively). Although the in vitro PC-3 cell uptake of [^18^F]AlF-NOTA_(5)_-MAL-FSH2 showed quite lower values than that of its FSH1 analog, a comparable PC-3 tumor uptake and a lower background were confirmed from both in vivo microPET images and ex vivo biodistribution studies. Compared with FSH1 derivative, liver and intestine uptake of [^18^F]AlF-NOTA_(5)_-MAL-FSH2 was significantly lower, but kidney uptake increased as a result of its functionalization by the GGGRDN linker. Considering its improved in vivo performances, evaluation of this vector in other tumor models, as well as its use in tumor-targeted therapy [122], could offer interesting perspectives.

Other membrane receptors like urokinase-type plasminogen activator receptor (uPAR) can be overexpressed and targeted in human prostate cancer [123]. Thus, Persson et al. described the [^18^F]AlF-labeling of a NOTA_(5)_-conjugated high-affinity uPAR-binding 9-mer peptide denoted AE105 [124]. Despite the very brief reaction time (5 min, 95 °C), the radiolabeling step reached quite high overall yields (>92.7%), probably due to the presence of 25% (*v/v*) ethanol in the reaction medium. The crude mixture required HPLC purification, nonetheless. In vitro stability of the radioconjugate was only determined in PBS after 30 min incubation and in vivo biodistribution studies in PC-3 tumor bearing mice showed a moderate bone uptake (3.54 ± 0.32%ID/g at 2.5 h p.i.), suggesting a slight in vivo degradation of [^18^F]AlF-NOTA_(5)_-AE105. This tracer remains of high interest since the ongoing of several large-scale clinical trials involving [^68^Ga]Ga-NOTA_(5)_-AE105 [125]. 

Glucagon-like peptide type 1 receptor (GLP-1r) is present on pancreatic beta-cells and is overexpressed in insulinoma, a neuroendocrine tumor of the pancreas [126]. Kiessewetter et al. have been interested in the development of peptide-based PET imaging agents for GLP-1r, notably through the design of [^18^F]AlF-labeled NOTA-MAL-extendin-4 analogs [127]. Both cys^0^ and cys^40^ isomers of extendin-4 were conjugated with NOTA_(5)_-monoethylmaleimide amide and labeled with aluminum [^18^F]fluoride, but only the Cys^40^ derivative was further evaluated. Despite a modest post-purification RCY for the radiolabeling step (23.6 ± 2.4% yield, which could have maybe been optimized by [^18^F]F^−^ concentration using a ion exchange cartridge and/or adding an organic co-solvent like ethanol or acetonitrile to the reaction medium), [^18^F]AlF-NOTA_(5)_-MAL-Cys^40^-extendin-4 proved to be stable in human plasma for up to 1 h, which is a critical parameter for GLP-1 analogs [128]. PET imaging of this radiotracer in insulinoma INS-1 xenografted mice showed good tumor uptake (15.7 ± 1.4%ID/g at 30 min p.i.) along with high radioactivity accumulation in the kidneys (79.25 ± 3.67%ID/g at 30 min p.i., which remains lower than that of [^111^In]In-DTPA- and [^68^Ga]Ga-DOTA-extendin-4 derivatives [129,130]) and very fast blood clearance.

Similarly, Xu et al. investigated the imaging properties of [^18^F]AlF-NOTA_(5)_-MAL-cys^39^-extendin-4, corresponding to Cys^40^ analog minus its Ser^39^ amino acid [131]. Radiochemical yields of the labeling step were once again quite modest (17.5 ± 3.2%, under almost identical reaction conditions to Kiessewetter et al.). However, the tracer displayed sufficient stability in human plasma (up to 180 min at 37 °C). Compared with the previous report, the tumor uptake of [^18^F]AlF-NOTA_(5)_-MAL-cys^39^-extendin-4 (9.15 ± 1.6%ID/g at 30 min p.i.) was slightly lower than that of the Cys^40^ derivative, with a comparable kidney uptake. However, the high tumor-to-background ratio is benefit for PET imaging of GLP-1r expression.

Wang et al. were interested in the development of a neurotensin receptor (NTR) PET probe starting from an octapeptide neurotensin analog Lys-NT20.3 [132]. Imaging of NTR, highly expressed in ductal pancreatic adenocarcinoma, could have significant benefits from both a prognostic and therapeutic perspective in NTR-positive cancers [133,134]. Since Lys-NT20.3 was conjugated with *p*-SCN-Bn-NOTA_(6)_, only moderate [^18^F]AlF-labeling yields were reached (30%), possibly because of the extra sixth valence offered by NOTA_(6)_. Fractions of the purified product were analyzed on HPLC at several time points, showing >90% radiochemical purity up to 4 h post-purification. However, no defluorination was detected at all time points, the major impurity being an isomer of the product. This interconvertible isomerization was also observed in other [^18^F]AlF-NOTA complexes and was confirmed by HPLC-mass spectroscopy [57,58,135]. [^18^F]AlF-NOTA_(6)_-Lys-NT20.3 was evaluated in vivo in AsPC-1 and Panc-1 tumor-bearing mice, displaying a relatively high tumor accumulation and good tumor-to-background contrast on both models, despite an apparently poor in vivo stability.

**Figure 11:**
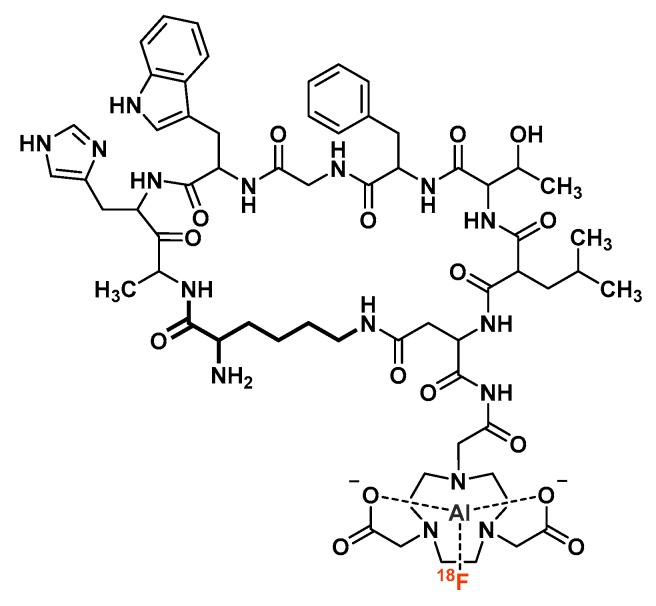
Chemical structure of [^18^F]AlF-NOTA_(5)_-C6.

Matrix metalloproteinases (MMPs) form a group of enzymes that can be associated with the metastatic potential of many neoplasias [136]. Liu et al. investigated PET imaging potential of a NOTA_(5)_-conjugated cyclic nonapeptide (C6) and selective MMP inhibitor [137]. [^18^F]AlF-labeling of this bioconjugate used conventional reaction conditions to obtain the expected product in fair to good yield (46.2–64.2%) and with a quite high molar activity (up to 48.3 GBq/µmol). Interestingly, [^18^F]AlF-NOTA(_5_)-C6 was highly stable in vitro (>95% after 4 h incubation, both in NaCl 0.9% and in human serum). Absence of radioactive degradation products may be partly due to the improved stability of the peptide, achieved via its particular cyclization mode (Figure 11) [138]. In vivo PET imaging study on SK-OV-3 and PC-3 tumor-bearing mice displayed favorable tumor radioactivity uptake and fast renal clearance of the tracer.

Li et al. evaluated [^18^F]AlF-NOTA_(5)_-G-TMTP1 as a PET probe in four different human hepatocellular carcinoma xenograft models [139]. Indeed, this linear hexapeptide displayed an important affinity for a series of highly metastatic tumor cells, though its binding mechanism is still unclear [140]. With a good in vitro serum stability (>95% after 2 h) and a high hydrophilicity (logP = −3.166 ± 0.022), microPET imaging study of this radiotracer showed an important accumulation in high metastatic potential xenografts (SMCC-7721 and HCCLM3). Meanwhile, tumor-to-muscle ratios in low metastatic potential hepatocellular carcinoma models (HepG2 and HCC97L) were almost three times lower. Furthermore, quite low activity in the liver and intestines was found in all models, confirming the potential of NOTA_(5)_-G-TMTP1 to target highly metastatic hepatocellular carcinoma, in both a diagnosis and therapy perspective.

WH701, a linear octapeptide identified by a phage-displayed library, is a specific ligand of TNFR1, a subtype of death receptor which is overexpressed in several varieties of carcinoma such as breast cancer [141]. Fu et al. conjugated the amino terminus of WH701 with NOTA_(5)_-NHS ester and labeled the expected bioconjugate with [^18^F]AlF [142]. Like [^18^F]AlF-NOTA_(5)_-G-TMTP1, [^18^F]AlF-NOTA_(5)_-WH701 appeared to be highly hydrophilic (logP = −3.07 ± 0.10) and was radiolabeled within 25 min in 38.1 ± 4.8% RCY. Tumor targeting properties of this tracer were evaluated in vivo in MCF-7 xenografts, after verification of the TNFR1 overexpression by this cell line via western blot. In addition to the tumor uptake, an evident uptake in inflammatory tissue could be observed, although lower (tumor/inflammation ratio = 1.34 ± 0.15). As expected from its high hydrophilicity, [^18^F]AlF-NOTA_(5)_-WH701 is characterized by predominant renal elimination and rapid blood clearance. As a perspective to improve its pharmacokinetic parameters, the authors suggest the conjugation of WH701 with PEG to improve its in vivo circulation time and tumor uptake.

Gu et al. synthesized a NOTA_(6)_-conjugate of a 7-mer peptide (IF7) targeting annexin 1, a highly specific surface marker of tumor vasculature [143]. NOTA_(6)_-IF7 was then radiolabeled with [^18^F]AlF. Purification protocol of this radioconjugate did not involve HPLC but only a C_18_ cartridge, allowing a short preparation time (20 min) along with a high RCY post-purification (92%). After the confirmation of its in vitro stability both in PBS and in mouse serum (>94% and 90.7% after 2 h, respectively), biodistribution studies of [^18^F]AlF-NOTA_(6)_-IF7 were performed in nude mice bearing A431 xenografts. The uptake of the stomach and small intestine were high at 30 min p.i. (22.09 ± 6.30 and 25.47 ± 9.16%ID/g, respectively) but decreased significantly by 60 min p.i., indicating that radiometabolites were excreted through the intestinal tract. It would have been interesting to determine logP of this compound to make a comparison with that of other [^18^F]AlF-labeled conjugates that are highly excreted through the kidneys. On microPET images after injection of [^18^F]AlF-NOTA_(6)_-IF7, tumors were clearly visible, with high contrast compared to the contralateral background and despite high radioactivity levels in the intestinal tract.

**Figure 12:**
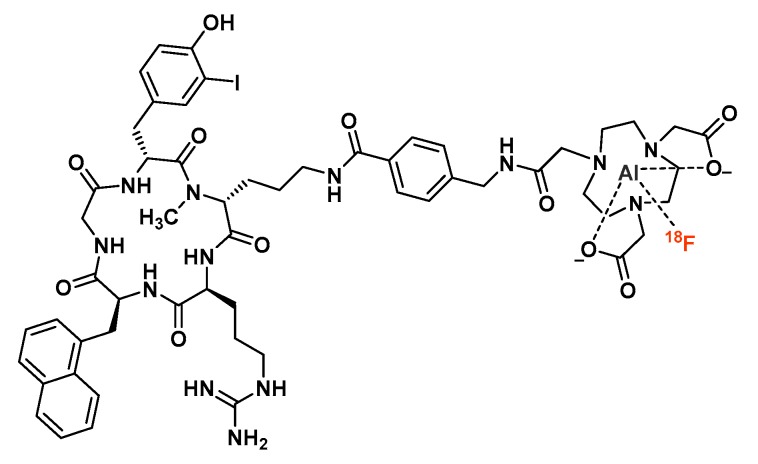
Chemical structure of [^18^F]AlF-NOTA_(5)_-Pentixather.

Several imaging probes have been developed to target the chemokine receptor CXCR4, which plays an important role in tumor aggressiveness, invasiveness and metastasis formation. Among these probes, pentixather is an original cyclic pentapeptide that displayed promising PET imaging properties when radiolabeled with ^68^Ga [144,145]. Poschenrieder et al. performed the [^18^F]AlF labeling of NOTA_(5)_-Pentixather (Figure 12), then studied its biodistribution and PET imaging properties in Daudi lymphoma-bearing mice [146]. Interestingly, labeling reaction conditions involved neither ethanol nor acetonitrile as a co-solvent, but dimethylsulfoxide (DMSO, 70% *v/v*). A sufficient RCY was therefore reached (45.6 ± 13.3%), then a C8 cartridge was used to purify the radiopeptide and remove DMSO. Compared to reference [^68^Ga]Ga-Pentixafor, [^18^F]AlF-NOTA_(5)_-Pentixather displayed a delayed in vivo blood clearance and higher uptake in non-target organs at 1 h post-injection. Due to the increased lipophilicity of [^18^F]AlF-NOTA_(5)_-Pentixather (logP = −1.4 vs −2.9 for its ^68^Ga-labeled analog), hepatobiliary excretion and non-specific accumulation in the liver were observed. Moreover, noticeable bone radioactivity levels may suggest a potential in vivo defluorination of the metal complex or the fixation on CXCR4-expressing hematopoietic cells. Tumor accumulation was almost identical for the two tracers, despite the improved in vitro CXCR4 affinity and the 3-fold increased internalization of [^18^F]AlF-NOTA_(5)_-Pentixather.

In the same way, Yan et al. described [^18^F]AlF radiolabeling of NOTA_(5)_-T140, a bioconjugate based on a 14-residues peptide that was found to possess potent CXCR4 antagonistic properties and high binding affinity [147]. Like [^18^F]AlF-NOTA_(5)_-Pentixather, this radiotracer demonstrated significant uptake in the bone and high uptake in the liver and kidney, which was probably not due to the CXCR4 expression but probably to the presence of metal chelators undergoing transchelation under physiological conditions [148]. Modulation of this tracer to optimize its biodistribution could be of high interest for purposes of its further evaluation.

#### 2.1.6. [^18^F]AlF-Labeled Peptides for Non-Oncological Applications

Clinical applications of PET imaging spread beyond the field of oncology and can be of interest in various areas, depending on the vector part of a radiotracer. Thus, several [^18^F]AlF-labeled probes were evaluated in non-oncological applications.

**Figure 13:**
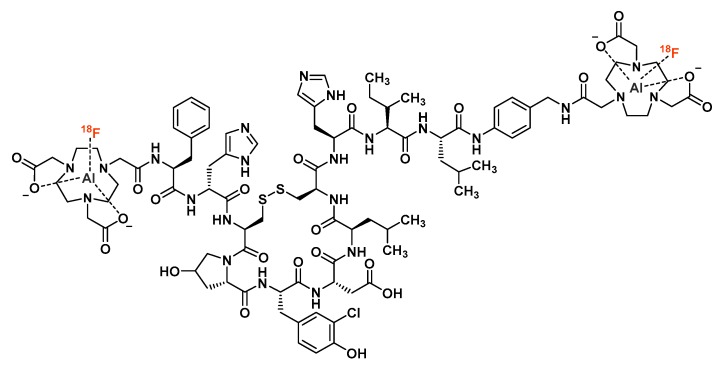
Chemical structure of bis-[^18^F]AlF-NOTA_(5)_ fibrin-binding positron emission tomography (PET) probe.

For PET imaging of thrombus formation, a process playing a prominent role in many cardiovascular disorders, Blasi et al. synthesized NODAGA- and NOTA_(5)_- bis-conjugated fibrin-targeting cyclic peptides (Figure 13) [149]. After being labeled with ^64^Cu or [^18^F]AlF in average to excellent yield, these tracers were investigated in a rat model of arterial thrombosis. The four probes were able to clearly detect thrombus from the background with minimal non-target uptake, 30- and 90-min post-injection. [^18^F]AlF radioconjugates, however, displayed lower target-to-background ratios and evident off-target signal in the bone. For most of the probes, liver uptake was approximately 50% lower than the thrombus while kidney uptake was about 10-times higher. Overall, these tracers demonstrated, along with a preserved affinity for fibrin, an improved metabolic stability (except for the [^18^F]AlF-NODAGA-peptide) in comparison with previously reported fibrin-specific radiopeptides [150]. This highlight points out the crucial influence that a suitable chelator can have on the in vivo fate of a radiotracer.

As a potential tracer for pulmonary circulation and pulmonary embolism imaging, Alonso Martinez et al. described the conjugation with NOTA_(5)_ and the formation of an [^18^F]AlF-complex with DFH17, a 31-amino acids PEGylated adrenomedullin analog [151]. In an extended study and optimization of the reaction conditions, the authors established that a low [^18^F]AlF-to-NOTA molar ratio (1:1 to 1:3) allowed the highest complexation yields. Addition of 50% ethanol (*v/v*) also greatly increased RCY of the crude labeling reaction, while high fluorine-18 activities (1110 to 2405 MBq) resulted in lower yields. A 6 mM AlCl_3_ concentration reduced RCY and optimal conditions were found for 2–3 mM AlCl_3_. [^18^F]AlF -DFH17 was finally obtained in moderate overall RCY (22–38%) in a rather long total preparation time of 63 min. PET imaging of [^18^F]AlF-DFH17 in normal Sprague-Dawley rats showed high tracer accumulation in the lungs up to 1 h p.i., along with rapid accumulation in kidneys. The absence of radioactivity in the bones suggested sufficient in vivo stability of the tracer.

Vascular adhesion protein 1 (VAP-1) is an endothelial glycoprotein involved in the transfer of circulatory leukocytes into tissues undergoing inflammatory responses. To bind this target and image inflammatory conditions, Moisio et al. used a fragment of sialic acid-binding immunoglobulin-like lectin 9 (Siglec-9), forming a 17-amino acids PEGylated cyclic peptide conjugated with NOTA_(5)_ [152]. [^68^Ga]Ga- and [^18^F]AlF-NOTA_(5)_-Siglec-9 radiocomplexes were thus synthesized and compared in vivo in inflammation-induced Sprague-Dawley rats. The [^18^F]AlF-labeled derivative was obtained in quite lower RCY than its ^68^Ga-labeled analog (39 ± 1% vs 64 ± 1%) in a two-times longer total synthesis time (60 min vs 30 min). With both tracers, dynamic PET imaging showed clear radioactivity accumulation in the inflamed tissues. According to the authors, the image quality of ^68^Ga- and [^18^F]AlF-labeled tracers was comparable, although radioactivity concentration in target tissues seemed slightly higher for [^18^F]AlF-NOTA_(5)_-Siglec-9.

Beard et al. were interested in the design of an [^18^F]AlF-labeled oxytocin receptor (OTR) tracer in order to objectify the nose-to-brain uptake following intranasal (i.n.) administration of oxytocin [153]. Thus, after a brief structure-activity relationships study based on a small library of peptide derivatives, an OTR-selective oxytocin analog (dLVT) was selected for conjugation with NODA and radiolabeling. Interestingly, the authors noticed that when the reaction mixture contained 50% *v/v* NaCl 20% (total reaction volume = 200 µL), used to enhance ^18^F elution from ion exchange cartridge, an increase in RCY was observed. This improvement was comparable to that obtained when organic co-solvent is added to the medium. When evaluated in vivo, [^18^F]AlF-NODA-dLVT showed an increased uptake in brain parenchyma after intranasal administration compared to i.v., though exposure remained low (0.11 ± 0.03%ID/g i.n. vs 0.05 ± 0.03%ID/g i.v.). Moreover, the tracer was unable to penetrate deeper regions of brain tissue during the timescale studied, suggesting that the i.n. route is not significantly more efficient than the i.v. route for this OTR-targeting tracer.

In order to examine the influence of age on GLP-1R expression in healthy rat brain, Wang et al. used the already described [^18^F]AlF-NOTA_(5)_-MAL-Cys^39^-Extendin-4 radiopeptidoconjugate as a possible imaging agent of neurological diseases [154]. Radiolabeling reaction conditions were strictly identical to those described by Xu [131], leading to [^18^F]AlF-NOTA_(5)_-MAL-Cys^39^-Extendin-4 in 20% RCY within 30 min. In vivo microPET imaging showed a slightly slower radioactivity accumulation in the brain of aged rats than in normal rats. Variations in the uptake of the tracer in different regions of the brain indicated that distribution of GLP-1R differs, depending on both on the region and on the age of rats. Like most of the peptidic PET probes, [^18^F]AlF-NOTA_(5)_-MAL-Cys^39^-Extendin-4 displayed important radioactivity uptake in kidneys that could harm its usefulness as a PET tracer.

### 2.2. [^18^F]AlF-Labeled Protein Conjugates

Many proteins of clinical interest are currently in use or subject to preclinical and clinical evaluation. This is particularly the case for monoclonal antibodies (MAbs) and MAb-derived macromolecules, including fragments, nanobodies and affibodies. In nuclear imaging, pharmacokinetic properties of the latter three types of biomolecules are better adapted than those of MAbs for short-lived elements radiolabeling. Indeed, the average molecular weight of these derivatives (around 6 kDa for affibodies, around 15 kDa for nanobodies and around 50 kDa for Fab and Fab’ fragments) allows quick biodistribution and attachment to their molecular target, along with a rapid clearance. On the other hand, MAbs are characterized by a delayed recognition of their target and, especially for human MAbs, a rather slow clearance. Hence, MAbs used in clinical nuclear imaging will be more likely to be labeled with long half-life radioelements, such as ^89^Zr (t_1/2_ = 78.4 h) or ^64^Cu (t_1/2_ = 12.7 h). Moreover, unlike most affibodies or nanobodies, entire MABs and fragments are heat-sensitive compounds and are thus unsuitable with [^18^F]AlF-labeling reaction conditions, systematically requiring a heating step to ~100 °C. Different solutions can be considered to address this disadvantage, such as the design of ligands allowing [^18^F]AlF chelation at room temperature or the chelator radiolabeling prior to its conjugation with the protein.

**Figure 14:**
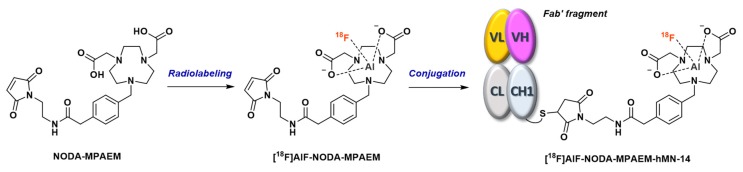
[^18^F]AlF radiolabeling and conjugation of NODA-MPAEM to a Fab’ fragment.

This second approach was first proposed by McBride et al. in 2012 [155]. A NODA-MPAA chelator, extended by a *N*-(2-aminoethyl)maleimide spacer (NODA-MPAEM, Figure 14), was first radiolabeled with [^18^F]AlF in classical reaction conditions (20 nmol ligand in 2 mM AcONa pH 4 buffer, 0.5 equiv. AlCl_3_, 730 MBq [^18^F]F^-^ in NaCl 0.9%, ~50% *v/v* acetonitrile, 415 µl total reaction volume), then conjugated to the Fab’ fragment of a humanized MN-14 anti-CEACAM5 IgG (Figure 14). Radiolabeled chelator was obtained in 82% RCY and the final radioconjugate was produced in 74% RCY after a 50 min total process. Biodistribution of the [^18^F]AlF-NODA-Fab’ radioconjugate was studied in Capan-1-xenografted mice and showed expected parameters for this type of tracer, like an elevated kidney uptake, low concentrations in blood and a favorable tumor-to-blood ratio (5.9 ± 1.3 at 3 h p.i.). Moreover, minimal bone uptake suggested that the [^18^F]AlF-NODA complex was very stable in vivo.

Following this proof of concept, Lütje et al. extended the previously described technique to the conjugation of the same radiocomplex ([^18^F]AlF-NODA-MPAEM) with other anti-CEA antibody derivatives such as a diabody or a dimeric fragment [156]. [^18^F]AlF radiocomplexes could be obtained in a quite higher labeling efficacy (94% ± 2%) and very good RCY for each radioconjugate (70% for reference [^18^F]AlF-NODA-Fab’ and 77% for both [^18^F]AlF-NODA-Diabody and dimeric fragment) within a <60 min total reaction time. Immunoreactivity of each radioconjugate was verified in vitro and was systematically conserved (>60%). In vivo, preferential localization of all [^18^F]AlF-radiofluorinated derivatives in CEA-expressing LS174T xenografts was observed, with a clearer visualization of CEA+ tumors with the Fab’ fragment and the dimeric fragment (tumor-to-blood ratio at 4 h p.i. = 5.87 ± 0.93 and 6.15 ± 1.58, respectively, vs 2.75 ± 0.18 for the diabody). Fab’ and dimeric fragment displayed a significant renal accumulation, contrary to the diabody, which was partly cleared via hepatobiliary route.

[^18^F]AlF-radiolabeled anti-HER2 affibodies have been particularly studied since the early years of this radiofluorination technique. Heskamp et al. reported in 2011 the 1-step [^18^F]AlF-labeling of NOTA_(5)_-Z_HER2:2395_ and its in vivo evaluation in SK-OV-3-xenografted mice [157]. This approach implied heating the NOTA_(5)_-conjugated affibody (90 °C, 15 min) to achieve modest radiolabeling yields (21.0% ± 5.7%). However, the final radioconjugate was isolated in >95% RCP and conserved its HER2-binding affinity. Imaging properties of [^18^F]AlF-NOTA_(5)_-Z_HER2:2395_ were compared to those of ^68^Ga- and ^111^In-labeled anti-HER2 affibody analogs, showing rapid clearance and high tumor-to-blood ratios for the PET derivatives. Low bone uptake indicated that this 1-step labeling protocol led to a stable complexation of [^18^F]AlF with the bioconjugate. According to the authors, [^18^F]AlF-NOTA_(5)_-Z_HER2:2395_ was preferred over its ^68^Ga-labeled analog for the longer half-life of ^18^F that allowed imaging several hours after injection, and therefore higher tumor-to-normal tissues ratios.

**Figure 15:**
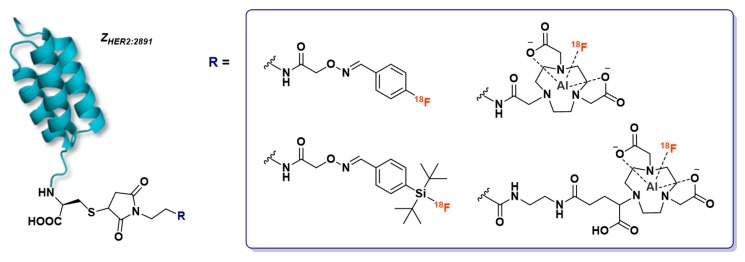
Schematic structures of ^18^F-radiolabeled Z_HER2:2891_ affibodies studied by Glaser et al.

Glaser et al. also described the 1-step radiofluorination of another anti-HER2 affibody (Z_HER2:2891_) following different approaches (Figure 15) such as silicon-fluoride acceptor, covalent link with 4-[^18^F]fluorobenzaldehyde and [^18^F]AlF complexation in NOTA_(5)_ or NODAGA [158]. The highest isolated RCY was reached with the Si[^18^F]FA-labeling technique (38% ± 2%), in a mixed aqueous/organic solvent medium heated at 95°C during 15 min. Covalently ^18^F-fluorinated affibody was obtained in 13% ± 3% within a longer reaction time (60–65 min vs 30–35 min for [^18^F]SiFA-labeling). NODAGA did not allow good [^18^F]AlF chelation (8% RCY, 72% radiochemical purity, 0.02 GBq/µmol molar activity) and was not studied further. NOTA_(5)_ was, as expected, a more suitable chelator, leading to 49% [^18^F]AlF incorporation but quite low isolated RCY (11% ± 4%). In vivo biodistribution and PET imaging in A431- and NCI-N87-xenografted mice (high and low HER2 expression, respectively) demonstrated the preferred pharmacokinetic profile of [^18^F]SiF- and covalently-labeled affibodies, whereas [^18^F]AlF-NOTA_(5)_-Z_HER2:2891_ displayed minimal differentiation between the two tumor types, higher kidney retention and increased bone uptake in time. Overall, this study suggests the potential interest to compare different radiofluorination methods for the same vector, in order to determine the influence of this parameter on the tracer pharmacokinetics.

With the same purpose of tumoral HER2 levels monitoring, Xu et al. used an anti-HER2 affibody (Z_HER2:342_) bearing a NOTA_(5)_ chelator at the end of a hydrophilic peptidic linker (GGGRDN, also used by Pan [111] and Zhu [120]) [159]. [^18^F]AlF radiolabeling reaction conditions, involving high [^18^F]fluoride activity (~3.7 GBq), around 0.3 equiv. AlCl_3_ and no organic co-solvent, allowed to obtain the expected radiocomplex within 30 min in 9.3% ± 1.5% RCY and >95% purity. The radiotracer was evaluated in vivo in high, medium and low HER2-expressing xenografted mouse models (SK-OV-3, JMT-1 and MCF-7, respectively). Interestingly, a significant linear correlation (R² = 0.99, *p* < 0.05) between %ID/g values and relative HER2 expression (measured by Western blot) was found for all tumor-bearing mice. Biodistribution results were in accordance with PET images, with a rapid localization in HER2-positive tumors, a prominent urinary excretion and a significantly reduced liver accumulation in comparison with the same covalently ^18^F-labeled affibody [160]. Reduced hepatobiliary excretion could be due to the increase in hydrophilicity conferred by the peptidic linker.

**Figure 16:**
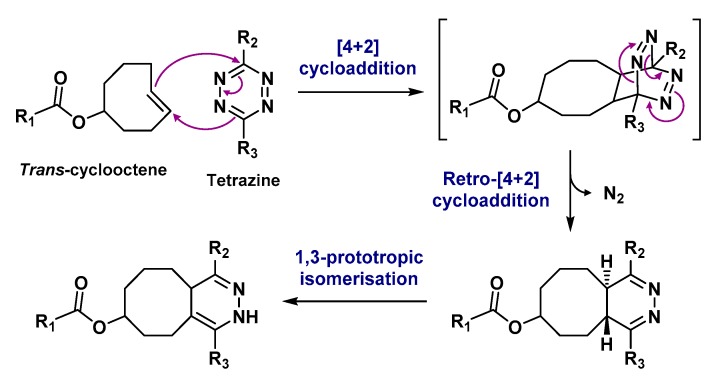
Inverse-electron demand Diels-alder reaction mechanism.

Since the renal accumulation of antibody-derived radiotracers can sometimes be considered as a significant drawback, Zhou et al. recently reported the design of an anti-HER2 single domain antibody fragment (sdAb 2Rs15d) functionalized with a renal brush border enzyme-cleavable glycine-lysine (GK) linker [161]. The 2-steps [^18^F]AlF radiolabeling protocol involved the formation of a [^18^F]AlF-NOTA_(5)_-PEG_3_-Tetrazine prosthetic group and its conjugation with sdAb 2Rs15d via a *trans*-cyclooctene (TCO)-tetrazine-based inverse electron demand Diels Alder (IEDDA) reaction (Figure 16). [^18^F]AlF-NOTA_(5)_-PEG_3_-Tz was obtained in 46.3% ± 4.1% RCY, in conventional reaction conditions. Conjugation was carried out in PBS at 20 °C and led to the expected radioconjugate in 17.8% ± 1.5% overall RCY, within 1.5 h. Noteworthy, this total preparation time could be shortened by avoiding the purification step which follows NOTA_(5)_-PEG_3_-Tz radiolabeling. Nevertheless, these results remained better than those obtained with other sdAb 2Rs15d radiofluorination strategies [162,163]. The in vivo biodistribution in healthy mice of the labeled sdAb showed a 15- and 28-fold lower renal uptake than that of its analog with no GK linker, at 1 h and 3 h p.i., demonstrating its significant interest. In SK-OV-3-xenografted mice, although the tumor uptake of the radioconjugate was lower than that seen for 2Rs15d labeled with ^18^F using other methods, tumor-to-kidney ratios obtained at 1 h were 1.5 to 10 times better.

As another HER receptor subtype, EGFR is an attractive target for both cancer molecular imaging and therapy. After the study of a covalently radiofluorinated anti-EGFR affibody (Z_EGFR:1907_) [164], Su et al. described 2 different radiofluorination strategies of Z_EGFR:1907_ [165]. The first approach consisted of conjugating the affibody with NOTA_(5)_ and radiolabeling with [^18^F]AlF. In a second way, the ^18^F-labeled 2-cyanobenzothiazole ([^18^F]FCBT) prosthetic group was conjugated to Z_EGFR:1907_. Due to a single purification step, [^18^F]AlF-NOTA_(5)_-Z_EGFR:1907_ total synthesis time was 3-times shorter than that of [^18^F]FCBZ-Z_EGFR:1907_ (40 min 1-step protocol and 120 min 2-steps protocol, respectively). However, both the overall RCY and molar activity of the [^18^F]FCBT derivative were considerably better (41% RCY, 22.2 GBq/µmol and 15% RCY, 1.5 GBq/µmol, respectively). Stability studies revealed significant differences between the two tracers, both in vitro and in vivo (>90%, 2 h in mouse serum and 90%, 1 h in vivo for the [^18^F]AlF derivative, 75%, 2 h in mouse serum and 40%, 1 h in vivo for the [^18^F]FCBT derivative). In vivo biodistribution of these two affibodies in A431 tumor-bearing mice was slightly different, demonstrating once again the noticeable influence of the labeling pathway on the tracer pharmacokinetics.

HER3 can also be overexpressed in a wide variety of cancers and could be predictive of tumor resistance [166]. Using the anti-HER3 affibody Z_HER3:8698_ as a vector, Da Pieve et al. compared the efficiency of 2 non-covalent radiofluorination techniques [167]. The first approach was a classic 1-step [^18^F]AlF chelation with a NOTA_(5)_-bioconjugate. The second method involved [^18^F]AlF complexation in a NODA-Tetrazine precursor, then its conjugation with cyclooctene-functionalized Z_HER3:8698_ via IEDDA reaction. [^18^F]AlF-NOTA_(5)_-Affibody was obtained in 38.8% ± 5.8% RCY and 6.0 to 11.9 GBq/µmol molar activity. Formation of byproducts, assumed to originate from the decomposition of the radioconjugate, was observed. The tetrazine-bearing [^18^F]AlF-NODA chelate was formed in very good yields (70% to 95%) and was engaged in the second step without further purification. Reaction with TCO-Z_HER3:8698_ yielded the product almost quantitatively. Two different IEDDA adducts could be identified: a dihydropyridazine and an aromatic pyridazine derivative. In vivo imaging properties of the two tracers in MCF-7 tumor-bearing mice were very similar, allowing high contrast images from 1 h post-injection. [^18^F]AlF-NODA-Z_HER3:8698_ displayed, however, slightly increased accumulation in intestine and in other non-target tissues, possibly due to its greater lipophilicity (logD = −1.87 ± 0.07, versus −2.19 ± 0.01 for [^18^F]AlF-NOTA-Z_HER3:8698_).

Considering the major clinical benefits of checkpoint inhibitors both in oncology and autoimmune diseases, Gonzalez-Trotter et al. designed a [^18^F]AlF-NOTA_(5)_-PD-L1-binding affibody as a PET tracer candidate [168]. [^18^F]AlF-NOTA_(5)_-Z_PD-L1_1_ was obtained in 15.1% ± 5.6% RCY, with a 14.6 ± 6.5 GBq/µmol molar activity and high RCP (96.7% ± 2%, following a preparative HPLC purification step). In vivo PET imaging after injection of this tracer demonstrated much higher tumor uptake in PD-L1-positive LOX tumor-bearing mice than in PD-L1-negative SUDHL6-xenografted mice (7:1%ID/g ratio at 90 min p.i.). Prominent renal clearance and retention were observed in all mice. These conclusive results suggest a possible clinical use of this type of tracer, whose value would be even greater if a kit formulation would be possible.

In the cardiology field, Lu et al. conjugated Cys-Annexin V with [^18^F]AlF-NOTA_(5)_-Maleimide with the view to target membrane phosphatidylserine exposed during apoptosis [169]. Through this 2-steps radiolabeling procedure, the final [^18^F]AlF-NOTA_(5)_-MAL-Cys-Annexin V was obtained within 65 min, with both high RCP and molar activity (97.38% ± 0.35% and 54.0 GBq/µmol, respectively). The conjugation step was achieved in 78.88% ± 5.23% RCY in PBS at room temperature, for an overall yield (from [^18^F]F^-^ to the radioconjugate) about 15%. After confirming the tracer was stable in vitro (>95% either in PBS, mouse serum and cell culture medium for 3 h), in vivo microPET imaging of apoptotic rat liver highlighted an increased liver uptake versus control (3.09 ± 0.08%ID/g and 0.50 ± 0.02%ID/g at 1 h p.i. respectively). As with most of other [^18^F]AlF-labeled tracers, [^18^F]AlF-NOTA_(5)_-MAL-Cys-Annexin V underwent high renal excretion (67%ID/g at 35 min p.i.), possibly due to the hydrophilicity of the [^18^F]AlF-NOTA_(5)_ complex. Overall, the authors emphasized the greater convenience of this non-covalent radiofluorination strategy compared to the use of other fluorinated prosthetic groups such as [^18^F]fluorobenzylethylmaleimide.

In order to develop an easy-to-label PET blood pool tracer, Basuli et al. studied the 2-steps [^18^F]AlF radiofluorinaton of rat serumalbumin (RSA) [170]. First, NODA-Bn-Tetrafluorophenyl ester (TFPE) was synthesized and labeled with [^18^F]AlF using DMSO as organic co-solvent (1:5 to 1:1 *v/v* in 0.5 M sodium acetate buffer, pH 5) to facilitate the reaction and properly solubilize chelator. Without any intermediate purification because of very high radiochemical conversion of [^18^F]AlF-NODA-Bn-TFPE (93% ± 5%), RSA was directly added to the reaction medium after cooling and pH adjustment to 8. After purification by size exclusion chromatography, the final radioconjugate was isolated in 45% ± 10% yield within 50 min. Rat biodistribution studies showed the highest retention of the tracer in blood up to 2 h p.i. and noticeable activity in lungs, heart and kidneys, proportionally to the specific plasma volume of these organs. PET images of rats allowed a good visualization of central vasculature, without any apparent activity in the skeleton. Pharmacokinetics of [^18^F]AlF-NODA-Bn-RSA appeared very similar to those of covalently labeled RSA with [^18^F]fluoronicotinate, suggesting that organ plasma volume measurements with these tracers give a valid estimation of these properties if carried out in early timepoints.

**Figure 17:**
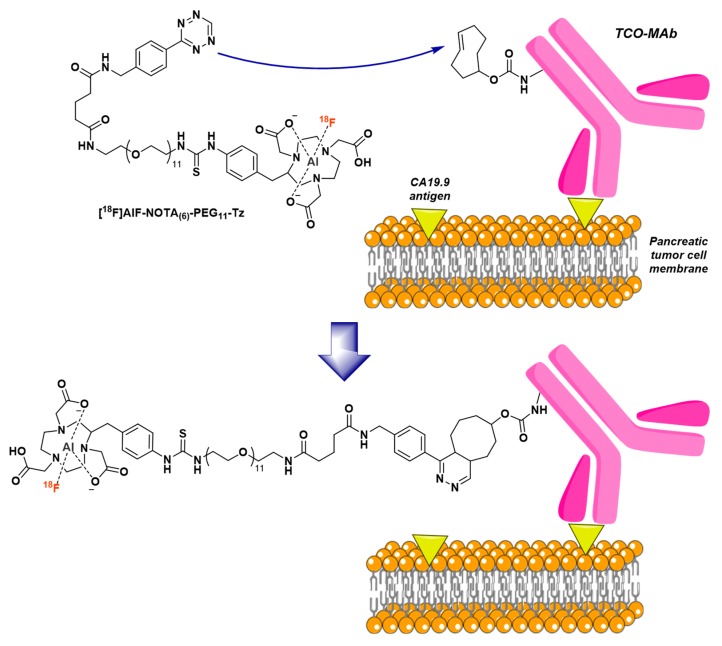
Pretargeting approach proposed by Meyer et al. involving an [^18^F]AlF-NOTA_(6)_-PEG_11_-Tz radioligand and a TCO-conjugated MAb.

In vivo bioorthogonal reactions are an original bioconjugation strategy which overcomes both antibody thermolability and its rather slow in vivo distribution step. In this kind of pretargeting approach, the antibody is radiolabeled within the body, after it has reached the tumor, facilitating the use of short-lived radioisotopes such as fluorine-18. Following this methodology, Meyer et al. described the Tz/TCO-based pretargeting of an anti-CA19.9 IgG with an [^18^F]AlF-NOTA_(6)_-PEG_11_-Tz radioligand (Figure 17) [171]. The [^18^F]AlF-NOTA complex was obtained in 54–65% RCY in high purity (>96%) and a molar activity between 21.4 and 26.7 GBq/µmol. It is worth noting that the quantities of organic co-solvent used are particularly significant (at least 3:1 MeCN/H_2_O). After validation of its in vitro and in vivo stability (79% ± 4.4% in human serum after 4 h, and 63% ± 8.9% in healthy mice after 4 h, respectively), biodistribution of [^18^F]AlF-NOTA_(6)_-PEG_11_-Tz alone was studied and showed in particular dual renal and fecal elimination pathways. Pretargeted biodistribution experiment on BxPC3-xenografted mice was conducted by injecting TCO-MAb 72 h prior to the radiolabeled probe and revealed increasing tumoral uptake over the course of the study. Uptake in other tissues remained low, except in the intestine and kidneys. PET imaging study was conducted the same way and confirmed the previous data, especially the increase over time of the tumor-to-background ratio. Optimization of the PK properties of the [^18^F]AlF-labeled probe in order to obtain high-quality images at earlier time points could be a major improvement of this strategy.

**Figure 18:**
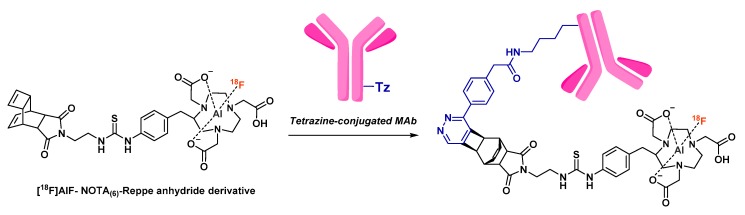
Pretargeting approach proposed by Shi et al. involving an [^18^F]AlF-NOTA_(6)_-Reppe anhydride derivative and Tz-conjugated MAbs.

Shi et al. employed a similar pretargeting protocol, with tetrazine-conjugated anti-EGFR MAbs and [^18^F]AlF-NOTA_(6)_-Reppe anhydride derivative (Figure 18) [172]. [^18^F]AlF-NOTA complex was synthesized in 30% RCY and >95% RCP after steric exclusion chromatography purification. Pretargeted biodistribution and metabolism study of [^18^F]AlF-NOTA_(6)_-Reppe anhydride displayed a quite comparable profile to that of [^18^F]AlF-NOTA_(6)_-PEG_11_-Tz [171], with a progressive HCT116 colorectal tumor uptake, a mixed hepatobiliary and renal elimination and a quite rapid decrease of the abdominal radioactivity. It should be noted that another advantage of this strategy over using long-lived radioisotopes is the substantially decreased overall radiation burden, especially on non-target tissues and organs.

**Figure 19:**
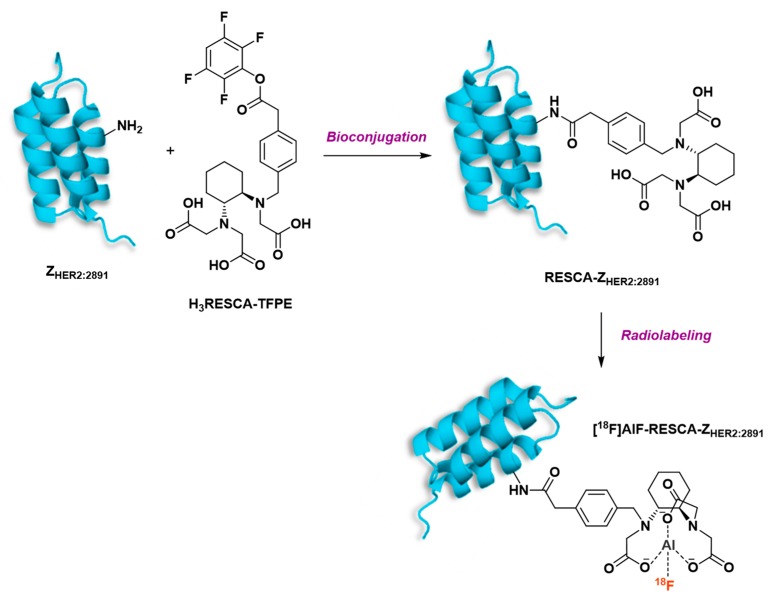
Conjugation and [^18^F]AlF-radiolabeling reactions reported by Cleeren et al. with original RESCA chelator.

To overcome the drawbacks associated with proteins thermosensitivity, Cleeren et al. designed an original restrained complexing agent (RESCA, see Figure 19) for application of the [^18^F]AlF radiolabeling strategy at room temperature [173]. H_3_RESCA-TFPE was first conjugated with lysine residues of three different proteins: human serumalbumin (HSA), anti-Kupffer cells nanobody and anti-HER2 affibody (Z_HER2:2891_). As a proof of concept, H_3_RESCA-HSA was directly labeled with [^18^F]AlF in good RCY (52% to 63%) and under mild reaction conditions (12 min, R.T., NaOAc 0.1 M pH 4.5). Final purification by size-exclusion chromathography provided the radioconjugate in high RCP (>98%) and within less than 30 min. Biodistribution study of [^18^F]AlF-RESCA-HSA in healthy rats confirmed the structural and functional integrity of the tracer, along with its good in vivo stability (only minor increase in bone uptake observed over time). Radiolabeling of anti-Kupffer cells nanobody at R.T. gave similar encouraging results (35% to 53% RCY, >98% RCP, <35 min synthesis time, 80 to 85 GBq/µmol molar activity), much better than those obtained with covalent radiofluorination protocols applied to the same kind of nanobody derivative [174,175,176]. However, purification of the crude residue needed 4 Hitrap desalting columns in series to isolate pure radioconjugate. As expected, this compound subjected to high renal accumulation and notable liver uptake in healthy mice. Moreover, bone uptake slightly increased over time, maybe because of a degradation/recycling cycle occurring in the kidney and releasing [^18^F][AlF]^2+^. Concerning affibodies, they are often considered heat-stable, however, thermal degradation of the radioconjugate is sometimes observed [167]. Thus, two batches of Z_HER2:2891_ were conjugated either with H_3_RESCA-Maleimide or NOTA-Maleimide. Then bioconjugates were [^18^F]AlF-labeled at R.T. (RESCA) or 100 °C (NOTA) with other reaction parameters remaining the same (15 min, pH 4, 50% *v/v* ethanol). [^18^F]AlF-RESCA-MAL-radioconjugate was obtained in slightly higher yields (20% ± 7% vs 8% ± 6%) but in same RCP, molar activity, and within the same reaction time. PET/CT study of these two radioconjugates in healthy male non-human primates showed very similar PK patterns for both, even though [^18^F]AlF-RESCA-MAL-Z_HER2:2891_ displayed quite lower radioactivity accumulation in kidneys because of a faster clearance to the bladder. Overall, RESCA chelator was presented as an appealing alternative to 2-steps protocols for [^18^F]AlF labeling of heat-sensitive molecules.

Following these findings, Cleeren et al. published a detailed protocol for both RESCA-nanobody coupling reaction and [^18^F]AlF radiolabeling step [177]. This overview highlights several critical reaction parameters, such as suitable pH (4.4 to 4.6 for a good [^18^F]AlF chelation), high molar activity [^18^F]F^-^ batch purified on ion exchange cartridge, optimal [^18^F]F^−^-to-Al^3+^ and chelator-to-Al^3+^ ratios (20 nmol AlCl_3_ for 1.1 to 2.2 GBq [^18^F]F^-^ and ~40 nmol chelator) or total reaction volume (~700 to 800 µL).

### 2.3. [^18^F]AlF-Labeled Small Molecule Conjugates

**Figure 20:**
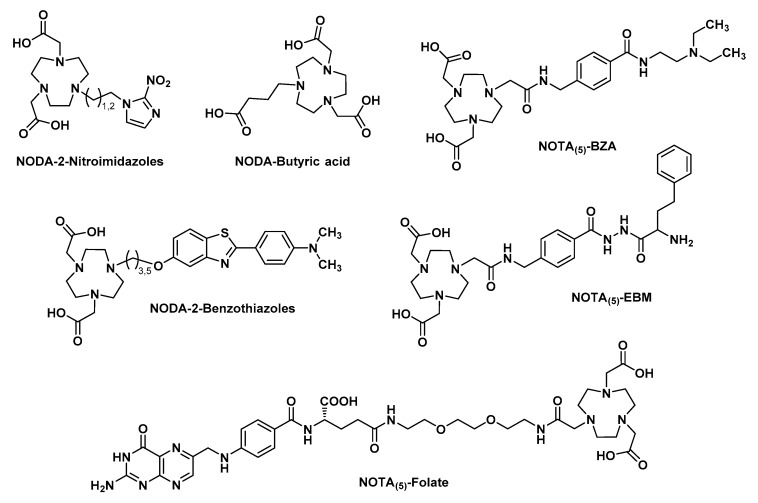
Chemical structures of several previously discussed [^18^F]AlF-labeled small molecules.

Functionalization of a small molecule with a bifunctional chelating agent towards its labeling with a radiometal remains a challenging technique, mainly due to the low permissiveness of these compounds to significant steric modifications. Although few in number (Figure 20), several small molecules bearing a chelating motif were labeled with [^18^F]AlF and evaluated as PET tracers.

By analogy with [^18^F]fluoromisonidazole, Hoigebazar et al. proposed the [^18^F]AlF radiolabeling of NODA-2-nitroimidazole derivatives for PET imaging of hypoxia [178]. Compounds with 2- or 3-carbons linker were synthesized and labeled in very good RCY (84.5% to 87.9%), high RCP (>99%), sufficient molar activity (3.9 to 4.3 GBq/µmol) and within only 15 min. Usual reaction conditions were used for this radiolabeling (0.1 M NaOAc pH 4, 45 nmol AlCl_3_, ~100 MBq [^18^F]^-^, 50 nmol vector, 110 °C, 10 min) and alumina cartridge followed by HPLC were necessary for the purification step. Both radiocomplexes showed very low in vitro plasma proteins binding (0.36% to 0.64% after 2 h incubation) and elevated in vitro uptakes on 3 cell lines under hypoxic versus normoxic conditions. Biodistribution and PET imaging studies in CT-26-xenografted mice evidenced early and predominant renal elimination along with better tumor-to-muscle and tumor-to-nontumor ratios at 1 h p.i. than those of reference drugs ([^18^F]FMISO and [^18^F]FAZA).

As a simple and original PET renal radiotracer, Lipowska et al. evaluated [^18^F]AlF-NODA-Butyric acid for kidney function imaging [179]. Radiolabeling conditions used were those published by Shetty et al. [56], with no organic co-solvent. RCY for the radiofluorination step is not specified, but the expected radiotracer was isolated after HPLC purification in >95% RCP. [^18^F]AlF-NODA-Butyric acid exhibited high in vitro and in vivo stability (>95%) over 4 h at physiological pH and at 15 min p.i., respectively. The [^18^F]AlF-labeled tracer, compared to *o*-[^131^I]iodohippurate, was highly specific for renal excretion and probably eliminated by both glomerular filtration and tubular secretion. Moreover, this specificity is conserved in a renal failure animal model, with only a small shift to hepatobiliary excretion, conferring to this molecule acceptable pharmacokinetic and chemical properties as a renal imaging agent.

Wang et al. developed a non-peptidic bivalent small molecular integrin α_V_β_3_-targeting compound (IA), conjugated with *p*-SCN-Bn-NODA and labeled with [^18^F]AlF for angiogenesis or metastatic potential imaging [180]. NODA-IA was radiolabeled in good RCY (65% to 75%) and high molar activity (27.75 to 31.45 GBq/µmol) after being easily purified on a C_18_ cartridge. In vivo evaluation of this tracer on two different xenografted mouse models showed a correlation between tumor uptake and integrin expression levels. In U-87 MG tumor-bearing mice (high integrin expression), [^18^F]AlF-NODA-IA showed rapid and high tumor accumulation (6.35 ± 0.67%ID/g at 1 h p.i.) along with a fast renal excretion. It would have been very elegant to directly compare the PET imaging properties of this tracer with a reference [^18^F]AlF-labeled RGD bivalent PET probe such as alfatide II.

Due to the specific binding ability of benzamide moiety to melanin [181], Chang et al. proposed an [^18^F]AlF-NOTA_(5)_-Benzamide derivative as a potent PET imaging agent of malignant melanoma [182]. After a 5-steps synthesis (~26% overall yield), NOTA_(5)_-Benzamide conjugate was radiolabeled with [^18^F]AlF, purified on HLB cartridge and isolated in 20–35% RCY within 40 min. In addition to a high in vitro stability (>95% in PBS and in human plasma after 2 h incubation), [^18^F]AlF-NOTA_(5)_-BZA displayed high cellular uptake in melanin-expressing cell lines and a conserved binding affinity to melanin. Biodistribution studies evidenced good tumor uptake and tumor-to-normal tissue ratios since 6 min p.i. in high melanin expressing B16F0-xenografted mice. Rapid radioactivity accumulation in kidneys and bladder suggested that [^18^F]AlF-NOTA_(5)_-BZA is mainly excreted via the urinary tract, which is consistent with its hydrosolubility (logP = −1.96 ± 0.14).

By analogy with flutemetamol, an ^18^F-fluorinated heterocyclic PET radiopharmaceutical targeting amyloid β (Aβ) plaques associated with Alzheimer disease, Song et al. synthesized NODA-conjugated 2-phenylbenzothiazole derivatives [183]. After the selection by in vitro binding assay of the molecule with the highest affinity for Aβ plaques, the retained compound was [^18^F]AlF-labeled within 40 min in 17.8% RCY and isolated in >98% RCP after HPLC purification. Ex vivo biodistribution study of this [^18^F]AlF-NODA-Benzothiazole tracer showed its initial brain uptake was significantly decreased compared with commercially available Aβ tracers (0.30 ± 0.04%ID/g at 2 min vs >4%ID/g at 2 min). However, in vitro autoradiography study pointed out the highly potent binding of this probe to Aβ deposits in blood vessels of cerebral amyloid angiopathy patients.

Fisher et al. evaluated a NOTA_(5)_-conjugated elastin-binding molecule (EBM) as a PET imaging agent for visualizing atherosclerotic plaque lesions [184]. Derived from previously published procedures [57,155], [^18^F]AlF-labeling of NOTA_(5)_-EBM was accomplished with up to 25% incorporation yield and 8% to 13% RCY after HPLC purification. The expected radioconjugate reached a quite high molar activity (59 ± 12 GBq/µmol). In vivo biodistribution study in healthy mice showed rapid radioactivity accumulation of [^18^F]AlF-NOTA_(5)_-EBM in the carotid arteries, heart and lung, known to have high elastin content. The tracer tends to be excreted through kidneys but also through the hepatobiliary system, despite its rather low logD_7.4_ (−1.3 ± 0.1). In vitro autoradiography studies on human carotid plaque sections found a higher binding of [^18^F]AlF-NOTA_(5)_-EBM in stable and vulnerable atherosclerosis plaques compared to normal human arteries samples. However, no clear differentiation between stable and unstable lesions was evident.

NOTA-folate radioconjugates were particularly studied in the recent years, both for cancer and non-oncological imaging. Silvola et al. investigated [^18^F]AlF-NOTA_(5)_-Folate for atherosclerotic plaque inflammation detection [185]. The radioconjugate was obtained in very good molar activity (77 ± 22 GBq/µmol) and high RCP (>95%) but within a rather long synthesis time (80 to 95 min), particularly because of semi-preparative HPLC purification step lasting 20 min. In order to stabilize [^18^F]AlF-NOTA_(5)_-Folate, the tracer was ultimately formulated in PBS (pH 7.4) in the presence of 8% ethanol and 4–7% propylene glycol, with a radioactive concentration kept under 400 MBq/mL at the end of the synthesis. Under these conditions, the tracer was stable for at least 4 h at room temperature. In vitro autoradiography on carotid endarterectomy samples from patients confirmed the colocalization of the tracer with folate receptor β-positive macrophages. Similarly, [^18^F]AlF-NOTA_(5)_-Folate administered in atherosclerotic mice and hyperlipidemic rabbits specifically accumulated in inflamed lesions, making it a promising tool for PET imaging of atherosclerosis plaques.

In the oncology field, the same tracer bearing either a PEG_2_ [186] or a PEG_12_ [187] linker was evaluated by Chen et al. for preclinical PET imaging of folate receptor-positive tumors. The overall radiosynthesis process was faster than described by Silvola [185] (~35 min), although radioconjugates were obtained in slightly lower RCY (8.4% to 18.6%). Biodistribution studies and microPET in vivo imaging in KB tumor-bearing mice demonstrated an important kidney uptake for the two tracers. Accumulation in the liver was considerably reduced by the introduction of a hydrophilic PEG_12_ linker. Considering its radiochemical and in vitro properties as well as its preclinical PET results, [^18^F]AlF-NOTA_(5)_-PEG_12_-Folate meets most of the requirements for possible translation in clinical use.

## 3. Current Clinical Use of [^18^F]AlF-Labeled Biomolecules

To date, very few [^18^F]AlF-labeled molecules were administered to man. These experimental radiopharmaceuticals may be subject to preconceptions because they contain aluminum, a metal that may accumulate in several organs and can be associated with bone, brain or liver toxicity [188]. With the aim of reducing aluminum exposure, especially from parenteral nutrition in preterm infants [189], the U.S. Food and Drug Administration (FDA) has set in 2004 the aluminum limit rate at 25 µg/L (≈ 927 nmol/L) for large volume parenterals. This limit is associated with the warning that repeated IV aluminum administrations >4–5 µg/kg/day (≈ 148–185 nmol/kg/day) could lead to central nervous system and bone toxicity [190]. Considering the classical [^18^F]AlF labeling reaction conditions, quantities of aluminum chloride rarely exceed a few dozen nanomoles in one multi-dose preparation (see Supplementary material). These very small quantities, combined with the punctual nature of a PET examination, are important arguments in favor of the non-toxicity of the aluminum traces contained in these radiopharmaceuticals.

As stated before, [^18^F]AlF-PSMA-BCH was evaluated on 11 patients with prostate cancer and displayed good detectability of tumor lesions with reasonable radiation exposure [101]. Nonetheless, the two [^18^F]AlF-labeled tracers that were the most studied in man are NOTA-PRGD_2_ derivatives alfatide I and alfatide II.

The first administration of alfatide I to human was in 2012 [191]. The vector was formulated in a kit, allowing the whole radiosynthesis process to be accomplished within 20 min, with 41.2% ± 2.0% RCY. Nine patients with primarily diagnosed lung cancer were examined by static and dynamic PET imaging with alfatide I. All tumors could be identified with this tracer, with mean standardized uptake values of 2.90 ± 0.10 at 1 h post-injection. Tumor-to-blood and tumor-to-muscle ratios were 2.71 ± 0.92 and 5.87 ± 2.02, respectively. This first study proved that alfatide I could specifically image α_V_β_3_ expression with good contrast in lung cancer patients and could be used for planning or response evaluation of therapies that affect angiogenesis. Other pilot studies came to the same conclusions in lung cancer [192,193,194] and glioblastoma patients [195]. Alfatide I also showed a potential interest in diagnosing metastatic lymph nodes in 13 patients with non-small cell lung cancer [196]. However, the diagnostic value of alfatide I in lymph nodes metastasis identification in 20 patients with differentiated thyroid cancer was stated as inferior to [^18^F]FDG [197]. Another study in 61 patients with esophageal squamous cell carcinoma found quite similar results but stated that alfatide I could still provide complementary molecular information about the metastasis of this cancer [198]. Recently, alfatide I PET imaging was shown to possibly be predictable of the response to antiangiogenic agent apatinib [199].

First evaluation of alfatide II in man was carried out in 2015, in 11 patients with bone metastases [200]. This study stated that alfatide II PET imaging could detect bone metastatic lesions with good contrast and higher sensitivity than [^18^F]FDG (92% and 77% positive rate, respectively). More specifically, alfatide II was superior to [^18^F]FDG in detecting osteoblastic and bone marrow metastases (70% vs 53% and 98% vs 77%, respectively). Quite similar results were highlighted in a series of 9 patients with brain metastatic lesions [201]. In contrast, a study on 44 patients suspected of primary breast cancer showed no superiority of alfatide II over [^18^F]FDG in identifying these lesions [202], although alfatide II PET imaging was of good performance in detecting strongly estrogen receptor-expressing lesions. Diagnostic value of alfatide II has also been investigated in lung inflammation patients (tuberculosis, sarcoidosis) and lung cancer patients [203]. Tracer uptake in malignant lesions was significantly higher than in tuberculosis (4.08 ± 1.51 vs 2.63 ± 1.34 SUVmax at 1 h p.i., respectively) and sarcoidosis lesions (three negative results), suggesting the value of alfatide II in the differentiation of inflammatory and cancerous lung diseases.

Overall, these pilot studies provided interesting preliminary results on the potential clinical uses of integrin α_V_β_3_ imaging by alfatide derivatives. In the future, studies with larger patient cohorts are warranted to formally decide on the clinical usefulness of these radiotracers.

## 4. Conclusions

Among the different radiofluorination methods available to label potential PET imaging agents, the non-covalent approach using aluminum [^18^F]fluoride has been widely exemplified since its first report in 2009. [^18^F]AlF radiofluorination can be applied to the labeling of various kinds of vectors such as peptides, small molecules or proteins, via a one- or two-steps protocol in aqueous medium. Indeed, this method has the advantage of bypassing the azeotropic drying step associated with covalent radiofluorination techniques. However, it may need an organic co-solvent to improve radiolabeling yields, precise monitoring of acido-basic conditions and a heating step at ~100 °C, especially unsuitable for direct thermosensitive molecules labeling. Numerous conjugates, in various application fields, have been successfully radiolabeled via [^18^F]AlF strategy and showed encouraging in vitro and preclinical potential. Three of them, fulfilling critical criteria such as in vivo stability, favorable PK parameters and high specificity, have reached clinical evaluation stages. The compatibility of these vectors with a kit formulation for direct [^18^F]AlF labeling and the possibility to automate this process further encourages their transfer to clinical use. Compared to ^68^Ga, synthesis of [^18^F]F is dependent on cyclotron production and may present routing constraints. In contrast, possibility of obtaining high activity [^18^F]F^−^ batches allows the preparation of high activity kits, usable for a large number of patients, when a conventional ^68^Ga generator eluate is only sufficient for three–four patients. Imaging properties of ^18^F are inherently better than those of ^68^Ga, nevertheless, the better quality of [^18^F]fluorine images compared to [^68^Ga]gallium seems only moderately notified. Overall, [^18^F]AlF radiofluoration strategy tends to provide a convenient, simple and fast way to label nine-membered ring conjugates with [^18^F]fluorine, opening both preclinical and clinical development perspectives for several PET radiotracers.

## Figures and Tables

**Table 1:** Physical properties of ^18^F and ^68^Ga.

Characteristics	Fluorine-18	Gallium-68
Formation	^18^O (p,n) ^18^F	^68^Ge → ^68^Ga
Half-life (h)	1.83	1.13
Maximal β+ energy (keV)	633.5	1899.1
Mean β+ energy (keV)	249.3	836.0
Positron emission rate (%)	96.9	89.1
Gamma photons emission: energy (keV) and rate (%)	None	578.5 (0.034); 805.8 (0.094); 1077.4 (3.22); 1261.1 (0.094); 1883.2 (0.137)

## Supplementary Materials

Supplementary Materials are available online. Table S1: [^18^F]AlF-labeled peptides reports in scientific literature. Table S2: [^18^F]AlF-labeled proteins reports in scientific literature. Table S3: [^18^F]AlF-labeled small molecules reports in scientific literature.
